# Genetic alterations of *Keap1* confers chemotherapeutic resistance through functional activation of *Nrf2* and Notch pathway in head and neck squamous cell carcinoma

**DOI:** 10.1038/s41419-022-05126-8

**Published:** 2022-08-09

**Authors:** Syed S. Islam, Khawlah Qassem, Shafiqul Islam, Rashed R. Parag, Mohammed Z. Rahman, Walid A. Farhat, Herman Yeger, Abdelilah Aboussekhra, Bedri Karakas, Abu Shadat M. Noman

**Affiliations:** 1 Department of Molecular Oncology, King Faisal Specialist Hospital and Research Centre, Riyadh, Saudi Arabia; 2 School of Medicine, Al-Faisal University, Riyadh, Saudi Arabia; 3 Department of Biochemistry and Molecular Biology, University of Chittagong, Chittagong, Bangladesh; 4 Department of Pathology, Chittagong Medical College and Hospital, Chittagong, Bangladesh; 5 Department of Urology, University of Wisconsin School of Medicine and Public Health, Madison, WI USA; 6 Developmental and Stem Cell Biology, Peter Gilgan Centre for Research and Learning, The Hospital for Sick Children, Toronto, ON Canada

**Keywords:** Oral cancer, Oral cancer

## Abstract

*Keap1* mutations regulate *Nrf2* activity and lead to chemoresistance in cancers. Yet the underlying molecular mechanisms of chemoresistance are poorly explored. By focusing and genotyping head and neck squamous cell carcinoma (HNSCC) that had available pathologic and clinical data, we provide evidence that *Keap1* displays frequent alterations (17%) in HNSCC. Functional loss of *Keap1* results in significant activation of *Nrf2* and promotes cancer cell growth, proliferation, and elevated cancer stem cell (CSCs) self-renewal efficiency and resistance to oxidative stress. Furthermore, decreased *Keap1* activity in these cells increased nuclear accumulation of *Nrf2* and activation of the Notch pathway, causing enhanced transcriptional alterations of antioxidants, xenobiotic metabolism enzymes, and resistance to chemotherapeutic treatment. Limiting the *Nrf2* activity by either *Keap1* complementation or by *Nrf2* silencing increased the sensitivity to chemotherapy in *Keap1*-mutated cells and repressed the CSC self-renewal activity. Our findings suggest that *Keap1* mutations define a distinct disease phenotype and the *Keap1-Nrf2* pathway is one of the leading molecular mechanisms for clinical chemotherapeutic resistance. Targeting this pathway may provide a potential and attractive personalized treatment strategy for overcoming chemotherapeutic resistance conferred by *Keap1* mutations.

## Introduction

Head and neck squamous cell carcinoma (HNSCC) is the utmost global health concern and affects >890,000 patients, and over 450,000 HNSCC-related deaths occur every year [1, 2]. To date, cisplatin (CDDP) and paclitaxel (PTX) based chemotherapy and radiotherapy remain the preferred and effective treatment options for advanced-stage HNSCC patients. Sadly, a proportion of patients do not respond to chemotherapy and develop resistance to treatment. The contributing factors to treatment failures due to therapeutic resistance are linked to cell apoptosis, drug efflux changes, DNA damage repair, and abundance of cancer stem cells (CSCs) [3, 4].

The Cancer Genome Atlas (TCGA) project has profiled an extensive landscape of somatic genomic alterations in HNSCC [5]. This revealed, as expected, that smoking-related HNSCCs have near-universal loss-of-function of TP53 mutations and CDKN2A inactivation with frequent copy number alterations. In addition to these mutations, several other pathway *Keap1*, and *Nrf2-* pathway mutations were found in patients with HNSCC.

Inactivation of Kelch-like ECH-associated protein-1 (*Keap1*) strongly induces NF-E2-related factor 2 (*Nrf2*), and acquires malignancy in several types of cancer [6]. *Nrf2-*associated oxidative stress plays a critical role in developing chemoresistance in various cancers such as; lung, breast, colon, and ovarian cancer [7–10]. Recurrent or metastatic HNSCC patients have a poor prognosis with less than 1-year median survival [11]. Platinum-based chemotherapy (cisplatin) has been established as a gold standard systemic agent in HNSCC [12]. However, cisplatin resistance is still a barrier to organ-sparing and the survival of patients with advanced-stage HNSCC. In addition, cisplatin treatment often enhances a fraction of putative head and neck cancer stem cells, which are highly tumorigenic in preclinical models of HNSCC [13]. This small population of CSCs that reside within HNSCC are relatively resistant to chemotherapies and are clinically predicted to contribute to tumor recurrence. Cisplatin-resistant tumor cells consist of a higher proportion of epithelial cell adhesion molecule (EpCAM) and induce the expression of IL-6 and tumorigenic cytokines that contribute to cisplatin-induced stemness [14]. Recently we have shown the *Nrf2-*associated mechanistic link by which HNSCC cells acquire therapeutic resistance to cisplatin [15]. We have also demonstrated reduced reactive oxygen species (ROS) activity *via Nrf2* activation. *Nrf2* activation is further concerted with interleukin-6 (IL-6) and p62 [14]. Ongoing evidence has reported that the *Keap1*-*Nrf2* pathway and levels of ROS (reactive oxygen species) contribute to the development of drug resistance and the progression of HNSCC [14]. In addition, recent evidence suggests that *Nrf2* and the Notch signaling pathway mutually function by regulating *Nrf2* downstream target genes and activating the Notch signaling, suggesting that the *Nrf2* and Notch signaling pathway play a critical role in cellular behaviors [16]. However, the effects of somatic alterations in *Keap1*-*Nrf2* on CSCs and chemoresistance have not been deeply explored.

In this study, we examined whether *Nrf2* pathway activation due to *Keap1* inactivation plays a role in HNSCC therapeutic resistance and CSC induction. Our results found that *Keap1* alterations caused *Nrf2* activation concerted with ROS suppression and have led to CSC induction and therapeutic resistance in HNSCC cells. Furthermore, downregulation of *Nrf2* activity enhanced chemotherapeutic drug sensitivity in *Keap1* mutated HNSCC cells. Our findings suggest that *Keap1* mutation status might be helpful for personalized treatment decision-making strategies for patients with HNSCC.

## Materials and methods

### Cell culture and patient samples

Human SCC9 [17] and Cal33 [18] head and neck cancer cell lines were previously described and purchased from American Type Cell Culture (ATCC, Manassas, VA). All cell lines were authenticated using a short tandem repeat analysis kit (Applied Biosystems, CA). Cells were cultured in DMEM supplemented with 10% fetal bovine serum (FBS) and 1% penicillin and streptomycin cocktail. Formalin-fixed paraffin-embedded tissues from 24 (*n* = 24) HNSCC patients treated in Chittagong Medical College Hospital (CMCH), Chittagong, were included in this study after obtaining full patient consent. The study protocol for the collection and use of patient tumor tissues and clinical information was approved by the Institutional Review Board at CMCH (052(1) 04-06-2014) and the King Faisal Specialist Hospital and Research Center (KFSH&RC; RAC# 2210031). We obtained patients’ informed consent following local and international regulations. Tumors were obtained from all consented patients at the time of surgery. Tumors were first minced and enzymatically dissociated with 2 mg/ml of dispase (Roche, USA) and then incubated with 0.25% Trypsin-ethylenediaminetetraacetic acid, passed through a 21-gauge syringe, and filtered through a 23 μm cell filter (Merck Millipore). Cells were either directly cultured in supplemented CSC medium or cryopreserved in 80% FBS and 20% dimethylsulfoxide until further use. The plasmid encoding *Keap1* pCMV-*Keap1* and pRGBp2 vectors were described previously [19, 20] and purchased from Origene Technologies (Rockville, Maryland, USA). The expression plasmid *Keap1* pCMV-*Keap1* was transfected into 293FT cells using transfection reagents. Post-transfection supernatants (after 24 h) were collected and used for infection. The medium was then replaced with the complete medium, and cells were grown for 3 days.

### *Keap1* and *Nrf2* DNA sequence analysis

#### Tumor DNA isolation

Genomic DNA was extracted from a total of 24 (*n* = 24) formaldehyde-fixed and paraffin-embedded (FFPE) head and neck tumor samples using QIAamp DNA FFPE Tissue Kit (Qiagen). Briefly, 2–3 tumor sections were treated with 1 ml xylene to remove paraffin, tissues were scraped and pooled and the tumor cell pellets were lysed after DNA precipitation, samples were loaded into columns. Following the column wash steps, DNA was eluted with 50 μl elution buffer, quantified, and stored at −20 °C for further analysis.

#### PCR amplification and Sanger sequencing

The coding regions of *Keap1* (ENST00000171111), and *Nrf2* (ENST00000397062) genes were PCR amplified using 20 ng DNA-specific primers containing M13 tail sequences in 25 μl reaction volume. Sequences for all the primers are provided in Supplementary Table S1. For tumor samples that did not have a sufficient amount of DNA, nested PCR amplifications were performed to obtain enough products for the Sanger sequencing reaction. High-fidelity *Taq polymerase* was used for PCR amplification to avoid errors during the amplification reactions. Samples with mutations were verified by repeating the PCR amplification and sequencing steps to rule out any PCR-related artifact. Both M13 forward and reverse primers were used for bidirectional sequencing of the amplified PCR products.

### *Keap1*, *Nrf2, Notch1, and Hes1* siRNA transfection and Notch inhibition by DAPT and cell proliferation assay

*Keap1* (siGENOME D-001210-01), *Nrf2* (siGENOME D-003755-01) SMARTpool, and non-targeting scrambled siRNA sequence (siGENOME D-001210-01) control pool was obtained from Dharmacon and described previously [14]. The ON-TARGETplus pool of Notch1 and Hes1 siRNA was obtained from Thermo Scientific. Transfection was performed in 50% confluent cells using Lipofectamine 200 (Invitrogen) and cultured in reduced serum medium OPTI-MEM following the manufacturer’s instructions. The specific siRNAs were transfected into SCC9 and Cal33 cells in triplicate.

### Measurement of ROS by DCFDA assay

Cells were preincubated with vehicle (control), CB-839 for 48 h and exposed to cisplatin treatment for 24 h. ROS level was measured using the 2’,7-dichlorofluorescein-diacetate DCFH-DA (Sigma; USA). Unless otherwise indicated, cells were treated with *Nrf2* siRNA and/or cisplatin for 72 h. Briefly, 1 mL of cell suspension was transferred to a 1.5 mL culture tube and 20 μM of DCFH-DA staining solution was added. Cells were gently mixed and incubated for 45–50 min at 37°C in the dark. Washed the cells and resuspended them in 400 μL of cold PBS. DCFH-DA fluorescence intensity was detected by flow cytometry, using the FITC channel on BD FACSAria flow cytometer (BD BioScience) and data were analyzed with FACSDiva software.

### Sphere forming assay

siRNA and scrambled-siRNA treated cells were cultured in six-well ultra-low attachment plates at a density of 1000 cells/well in a growth factor-supplemented CSC medium. The number and size of the spheres were monitored and recorded every 3rd day. Sphere forming efficiency was calculated as the number of actual spheres/number of cells plated × 100.

### AlamarBlue cytotoxicity and proliferation assay

Cells were seeded (5000 cells/well) in a 96-well plate in a complete medium. Cells were treated with an increasing concentration of cisplatin alone or a combination of CB-839 for 72 h as indicated concentrations in the figure legends. Cell viability was assessed by AlamarBlue assay using the manufacturer’s instructions. AlamarBlue was added (10% of total volume) and incubated for 4 h in an incubator and fluorescence was measured using the SPECTRAmax Gemini Spectrophotometer (540 nm excitation and 590 nm emission). DRC (Dose-response curve) and GRmetrics packages were used to generate dose–response curves using R-Statistical software (Version 4.0.3). Inhibitory EC50 concentration values were calculated (DRC and GRmetrix package) from the results of cisplatin concentrations in triplicate from three independent experiments.

### Quantitative real-time (qRT-PCR)

Total RNA was extracted from fresh tumor tissues, SCC9, and Cal33 cells using RNAeasy Kit (Qiagen) and reversed transcribed. Total RNA was isolated from formalin-fixed, paraffin-embedded tumor tissue sections using RNAeasy FFPE kit (Qiagen) and reverse transcribed. SYBR-Green-1-based RT-PCR amplification was performed in triplicates on the LightCycler-480 (Roche). The primers list is shown in Supplementary Table S2. The relative expression of each gene was analyzed by comparing its expression to that of GAPDH.

### Western blotting

Lysed protein was transferred to the PVDF membrane and primary antibodies were added to PVDF membranes in 5% non-fat dry milk in TBS-Tween-20 buffer. Primary antibodies are *Nrf2* (Abcam, cat# ab137550, MA, USA) and *Keap1* (Abcam, cat# 119403 MA, USA), Notch1 (cat# 14-5785-81) and Hes1 (cat# PA5-28802; Invitrogen, USA), and GAPDH (Santa Cruz, cat# sc32233, CA, USA). HRP-conjugated anti-mouse or anti-rabbit secondary antibodies were used for the detection.

### Immunohistochemistry

The following primary antibodies against *Nrf2* (Abcam, cat# ab137550, MA, USA), *Keap1* (cat# ab119403, Abcam, MA, USA), CD44 (Abcam, cat# ab6124, MA, USA), *TP53* (cat# ab238069, Abcam, cat# ab238069, MA, USA) Notch1 (cat# 14-5785-81, Invitrogen, USA), and Hes1(cat# PA5-28802, Invitrogen, USA) were applied on deparaffinized 5-μm thick formalin-fixed tissue sections for overnight. A horseradish peroxidase (HRP)-conjugated secondary antibody was used for the detection. For *Nrf2* detection in cells, only nuclear immunostaining was included in this study because only transcriptionally active *Nrf2* resides in the nucleus.

### Notch inhibition and proliferation assay

ON-TARGETplus Pool of siRNA against Notch1 and Hes1 and non-targeting Pool of siRNA (Thermo Scientific) were used to downregulate the expression of Notch1 and Hes1 or used as a control in the experiments. Cells were seeded in 96-well plates in a complete medium and allowed to grow up to 70% confluence. Cells were transfected with siRNA using Lipofectamine RNAiMAX Reagent (Invitrogen). AlamarBlue was added (10% of total volume) and incubated for 4 h in an incubator and fluorescence was measured using the SPECTRAmax Gemini Spectrophotometer (540 nm excitation and 590 nm emission). Notch1 was inhibited with DAPT, a gamma-secretase inhibitor.

### Computational analysis of TCGA datasets

We downloaded the head and neck cancer RNA-Seq data set (Illumina HiSeq 2000) from the Cancer Genomic Atlas (TCGA, https://portal.gdc.cancer.gov), and analyzed using R-statistical software (Version 4.0.3).

### Statistical analysis

Experiments were performed in triplicates where necessary and results were presented as mean ± SEM. For independent data with two specimens, a two-tailed *t* test for equal variance, or one-way ANOVA Tukey post hoc comparison for three or more groups were applied. For all statistical analysis we used “R” statistical software (version 4.0.3), and for graphs “ggplot2” packages in “R”. Kaplan–Meier survival curves were generated and analyzed using “R” packages “survival” and “survminer”. The significance was calculated using Log-rank and Mantel-Cox test. Dose–response was analyzed using a DRC (Dose–response curve) and GRmetrix packages in “R” statistical software (version 4.0.3). The significance was defined based on *P* < 0.05.

## Results

### *Keap1* and *Nrf2* mutations predict shorter overall survival in patients with advanced HNSCC

**Fig. 1:**
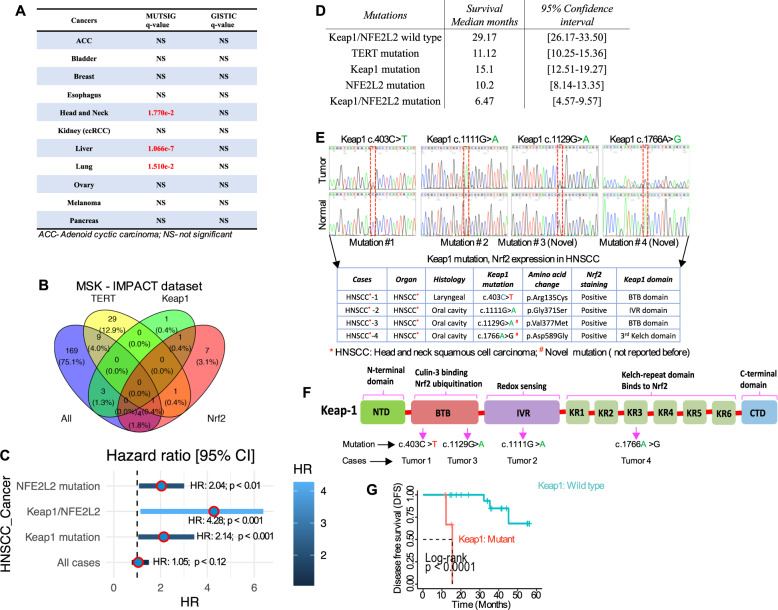
*Keap1*/*NFE2L2 (Nrf2)* mutations predict shorter overall survival in patients with HNSCC. **A** Alterations of the *Keap1* gene in major cancer types from TCGA database. **B** Venn diagram indicates the number of patients with head and neck cancer in the MISK-IMPACT database that is wild-type for the mutation (green), mutant for TERT (yellow), mutant for *Keap1*(light blue), and mutant for *Nrf2* (purple) (*n* = 186). **C** Multivariate Cox regression analysis for each indicated variable was performed. **D** The risk ratio of overall survival corresponds to each indicated variable. *Nrf2* (*P* < 0.01), *Keap1/Nrf2* (*p* < 0.001) and *Keap1* (*p* < 0.001) mutations are independently identified as significant covariate for overall survival. The table indicates the overall survival across each group with a 95% confidence interval. **E** Electropherogram depicting *Keap1* mutation sequence analysis for HNSCC. The top part shows the detection of the *Keap1* mutations identified in HNSCC patients’ tumors and the bottom part shows the non-cancerous normal individuals’ *Keap1* sequence. The table below shows the details of each patient and amino acid changes and corresponding *Nrf2* positivity. **F** Schematic diagram of conserved domain showing the structure of *Keap1* protein and the location of each mutation within *Keap1* protein. NTD N-terminal domain (amino acids), BTB broad complex-Tramtrack-Bric-a-brack, IVR intervening regions, KR Kelch repeat. **G** Kaplan–Meier disease-free survival analysis curve of *Keap1* wild-type and *Keap1* mutant HNSCC patients (*n* = 24; 4-*Keap1* mutant and 20- *Keap1* wild-type patient) (Log-rank *p* < 0.0001).

*Keap1* mutations and the resulting *Nrf2* activations have been reported in many cancers [5, 21]. As an approach to exploring the tumor-associated *Keap1* alterations, resulting in *Nrf2* activation and chemotherapeutic resistance through CSC induction, we first investigated the presence of genomic alterations of *Keap1* in a large panel of 21 distinctive cancers sequenced by The Cancer Genomic Atlas consortium (TCGA) and recently developed mutations significance method (MutSigCV), which provides a statistical metric to identify driver candidates in cancer with respect to the gene nucleotide length and the background mutations rate of each type of cancer analyzed [5, 22]. This analysis revealed that the *Keap1* mutations occurred in several cancers, including head and neck cancer (Fig. 1A; Suppl. Fig. 1A). Memorial-Sloan Kettering-Integrated Mutation Profiling of Actionable Cancer Targets (MSK-IMPACT) is a platform for archiving a hybridization capture-based next-generation sequencing panel that detects protein-coding mutations and copy number alterations (CNAs) and selects promoter mutations and structural rearrangements in more than 410 cancer-associated genes [23, 24]. We explored a cohort of 186 sequentially profiled HNSCC patients for tumor-specific somatic mutation in *Keap1* only (*n* = 1), *Nrf2* only (*n* = 7), or both (*n* = 1) (Fig. 1B). Additionally, we included a third mutation in our analysis, TERT (*n* = 40), since TERT mutations often co-occur with *Nrf2* (*n* = 1), but not with *Keap1* (*n* = 0). In this cohort, we observed a marked increase in the hazard ratio (HR 4.28, *p* < 0.001) and a significant decrease in median survival from 29.17 months in patients with *Keap1* and or *Nrf2* wild-type (WT) to 15.1, 10.2, and 6.47-months patients harboring either *Keap1* and or *Nrf2* alone or double mutations (Fig. 1C, D). In a multivariate analysis, *Keap1* and *Nrf2* double mutations significantly (*p* < 0.001) predicted overall poor survival (Fig. 1D). In the TCGA data set, *Keap1* gene alterations are mostly missense mutations that occur in Kelch or BTB domains of *Keap1* (Suppl. Fig. 1B), thereby impeding *Keap1* protein interaction with *Nrf2*. On the other hand, *Nrf2* mutations are mostly missense and occur within the first 100 amino acids that contain the Neh2 domain. That includes the two degrons bound by the *Keap1* and thus likely impede *Keap1*-mediated *Nrf2* degradation [25] (Suppl. Fig. 1C).

To explore the role of *Keap1* mutations in HNSCC pathogenesis, and given the size of the available clinical samples, we begin by examining the *Keap1* mutations in our samples (*n* = 24). We amplified and sequenced all five protein-coding exons of the *Keap1* from 24 HNSCC surgical samples. We found 4 (17%) *Keap1* mutations and 2 out of 4 mutations had novel pathogenic somatic *Keap1* mutations (c.403 C > T and c.1129 G > A), while the remaining two mutations (c.1112 G > A and c.1766A > G) had likely pathogenic and benign germline in nature (Fig. 1E). Intriguingly, tumors with *Keap1* mutations showed positive *Nrf2* expression (Fig. 1E; the table below). These mutations reside in the functionally important *Keap1* protein domain, such as BTB, IVR, and KR regions, governing *Nrf2* ubiquitination, redox sensing, and *Nrf2* binding sites (Fig. 1F). The significance of mutations was further analyzed by in silico predictors (Suppl. Table 3). We also detected five separate synonymous germline variants in various frequencies (Suppl. Table 4). The most notable variant is *Keap1* c.1815G > A and is highly enriched in the HNSCC population compared to previously reported global healthy population frequencies. Prognostic analysis of patient tumors carrying *Keap1* mutations revealed a significant correlation with poor DFS (*p* < 0.0001 by Log-rank analysis; Fig. 1G). Due to frequent alteration of *Keap1* and *TP53* [26] in HNSCC, we were interested in whether *Keap1* alterations showed association with *TP53* molecular alterations. As expected, frequent *TP53* overexpression (12/24, 50.0%) was detected in our cohort. Interestingly, *Keap1* alterations were detected exclusively in the *TP53*-overexpressed HNSCC tumors (Suppl. Table 5).

### *Keap1* mRNA expression and concurrent *Keap1* and *Nrf2* mutations in *Nrf2* immunopositive HNSCC tumors

**Fig. 2:**
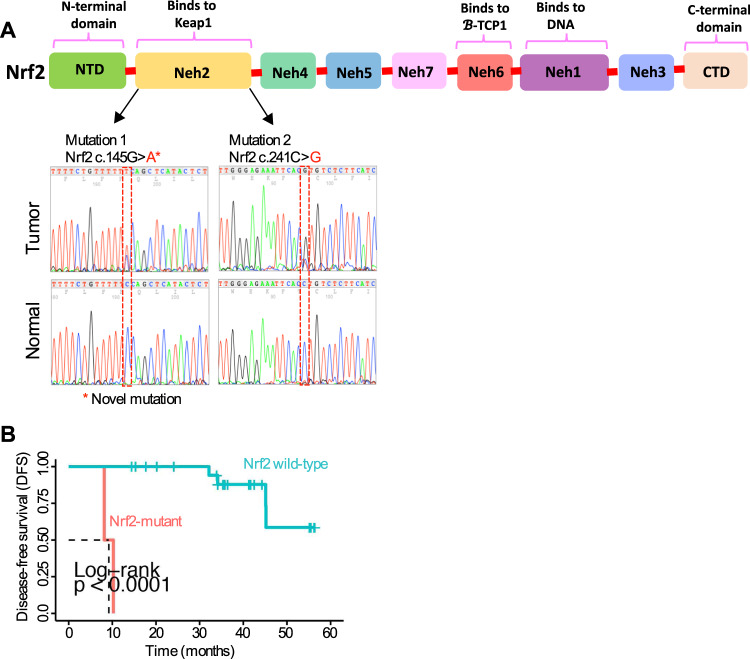
*Keap1* mRNA expression and concurrent *Keap1*/*Nrf2* mutations in *Nrf2* immunopositive HNSCC tumors. **A** Electropherogram depicting *Nrf2* mutation sequence analysis for head and neck cancer. The top part shows the locations of each mutation within the *Nrf2* protein. The bottom part shows the detection of *Nrf2* mutation identified in HNSCC patients’ tumors in non-cancerous normal individuals in the *Nrf2* sequence. **B** Kaplan–Meier disease-free survival analysis curve of *Nrf2* wild-type and mutant HNSCC patients (*n* = 24, 2-*Nrf2* mutant and 22 *Nrf2* wild-type patient) (Log-rank *p* < 0.0001).

*Keap1* is an essential regulator of *Nrf2* functions, and the role of *Keap1* in regulating *Nrf2* signaling in cancers has been reported previously [27]. To evaluate *Keap1* expression and concurrent mutations of *Keap1* and *Nrf2* in *Nrf2* immunopositive tumors, we first analyzed *Keap1* mRNA expression by qRT-PCR, followed by *Nrf2* sequence analysis in *Nrf2* immunopositive tumors (*n* = 24). As shown in Supplementary Table S6, 4 tumors with positive nuclear *Nrf2* staining had absent *Keap1* transcript expression, with the remaining 19 tumors being positive for *Keap1* transcript. Notably, although, one tumor had absent *Keap1* transcript expression (HNSCC-17, oral cavity, Suppl. Table 6) but harbored no *Keap1* mutations and was also positive for *Nrf2* protein expression. We then sequenced the *Nrf2* gene from all tumor samples that had positive *Nrf2* staining (Fig. 2A). Somatic *Nrf2* mutations were found only in 2 tumors (c.145 G > A and c.241 G > C) including a novel *Nrf2* mutation (c.145 G > A) in the Neh2 domain where *Keap1* binds and with high cytoplasmic *Keap1* expression (Fig. 2A; Suppl. Table 3). In our samples, no tumors harbored both *Keap1* and *Nrf2* mutations concurrently, confirming the MISK-IMPACT results in Fig. 1B. Given the smaller size of the available clinical samples, however, prognostic analysis of HNSCC carrying a *Nrf2* mutation revealed a significant correlation with poor DFS (~ 10 months) (*p* < 0.0001 by Log-rank analysis; Fig. 2B, Suppl. Fig. 2).

### The biological effect of *Keap1* mutations and effects of glutaminase inhibitor CB-839 in chemosensitizing *Keap1* mutant cells

**Fig. 3:**
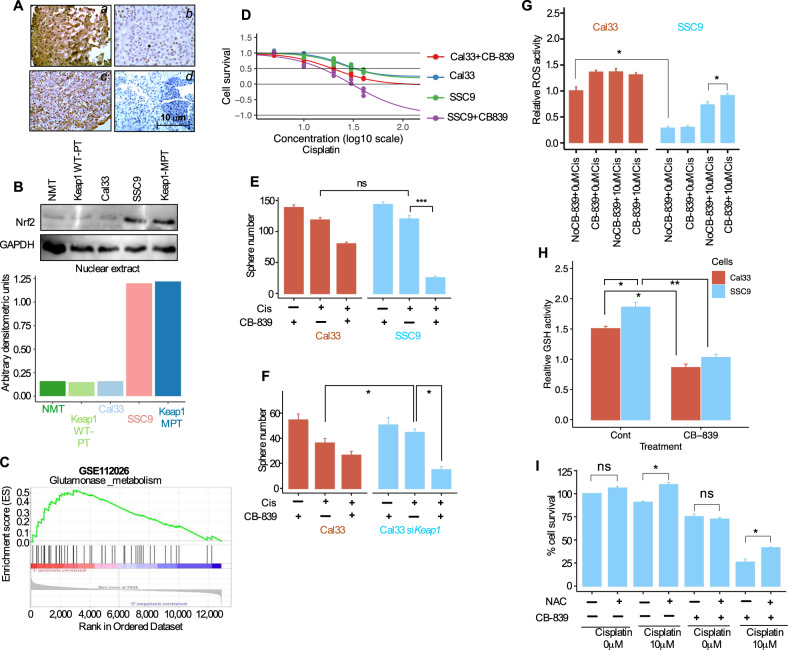
The biological effect of *Keap1* mutations and *Nrf2* overexpression in altered HNSCC tumor cells. **A** Immunohistochemical assessment of *Nrf2* expression in HNSCC tumor tissues. Part ‘a’ shows the strong nuclear and cytoplasmic *Nrf2* expression in the *Keap1* mutated patient (patient #3). Part ‘b’ and ‘c’ shows comparatively weaker cytoplasmic and nuclear *Nrf2* expression in *Keap1*-wild-type tissue. Part ‘d’ show negative Nrf2 staining in adjacent normal tissue. **B** Immunoblot analysis of *Nrf2* from nuclear protein in patient’s tumor cells, HNSCC cell lines, and non-malignant tissue. (bottom: Quantification of *Nrf*2 protein band density after normalizing with GAPDH). NMT: Non-malignant tissue; *Keap1*-WT-PT: *Keap1* wild-type patient tumor; *Keap1*-MPT: *Keap1* mutant patient tumor. **C** Gene set enrichment analysis (GSEA) of previously defined glutamine metabolism signature using RNA-seq data from GSE112026 data set. **D** Cell survival of Cal33 *(Keap1* wild-type) and SSC9 (*Keap1* mutant) cells with or without CB-839 (100 nM, 24-hour pre-treatment) and cisplatin (*n* = 3). Results were normalized with untreated cells. **E** Relative number of Spheres of Cal33-*Keap1* wild-type and *Keap1*-mutant SSC9 cells with or without CB-839 (100 nM, 24-hour pre-treatment) and cisplatin (*n* = 3, 10 μM, *n* = 3, ***P < 0.001). **F** Relative number of Spheres of *Keap1* wild-type Cal33 cells with or without knockdown of *Keap1* by *Keap1* specific siRNA in the presence or absence of CB-839 (*n* = 3, 100 nM, 24-hour pre-treatment) and cisplatin (10 μM, *n* = 3, *P < 0.05). **G** Intracellular reactive oxygen (ROS) levels measured by DCFDA intensity via FACS in Cal33 (*Keap1* wild-type) and SSC9 (Keap1 mutant) cells with or without CB-839 and cisplatin treatment (*n* = 3, 10 μM, *P < 0.05). Results were normalized with untreated cells. **H** GHS (Glutathione) activity analysis of Cal33 (*Keap1* wild-type) and *SSC9 (Keap1 mutant)* cells with or without CB-839 (*n* = 3, 10 μM, *P < 0.05, **P < 0.01). **I** Cell survival of SSC9 *Keap1* mutant cells treated in the absence or presence of cisplatin (10 μM) or CB-839 and with or without NAC (*n* = 3, *P* < 0.05).

To evaluate the loss of *Keap1* and its effects on *Nrf2* overexpression and cellular localization in primary HNSCC tumors, we immunoassayed *Nrf2* expression using the anti-*Nrf2* antibody in HNSCC primary tumor tissues. Strong nuclear and cytoplasmic and nuclear *Nrf2* expression was detected in primary tumor tissues harboring *Keap1* mutations (Fig. 3A; part a). Five tumor tissues with wild-type *Keap1* demonstrated decreased nuclear *Nrf2* and weak cytoplasmic *Nrf2* expression (Fig. 3A; parts b, c), while the normal tissue did not show *Nrf2* expression (Fig. 3A, part d). We also detected nuclear *Nrf2* localization in parts of *Keap1*-wild-type tumor tissues but to a lesser extent than *Keap1*- mutant tissue (Fig. 3A, part c). Aiming to study the levels of known *Nrf2* target genes in tumor and normal samples, we measured the total GSH levels and enzymatic activity of SOD1, NQO1, and GST levels in eleven primary tumors and adjacent normal tissues. Among all tissues, four tumors (4/11; 36%) harbored *Keap1* mutations, 2 (2/11; 18%) *Nrf2* mutations, and remaining samples (5/11; 45%) from patients with *Keap1* wild-type status (Suppl. Fig. 3A). We examined the GSH, SOD1, and NQO1 enzyme activity and GST levels in tumor and normal cells and found that these enzymes were at relatively higher levels in tumor tissues than in their corresponding adjacent normal tissues. Importantly, patients carrying *Keap1* and *Nrf2* mutations had higher levels of GSH, SOD1, NQO1, and GST compared to wild-type (Suppl. Fig. 3A).

To determine the nuclear accumulation of *Nrf2*, we immunostained the *Nrf2* protein in *Keap1* mutant SSC9 cells. The results showed nuclear accumulation of *Nrf2* protein in *Keap1-* mutated SSC9 cells (Suppl. Fig. 3B). To further examine the nuclear accumulation of *Nrf2*, we immunoassayed *Nrf2* in *Keap1* mutant and WT patients’ tumor samples and in two established HNSCC cell lines. Tumor cells with *Keap1* mutations (SSC9 and *Keap1*-mutated patient’s tumor cells) demonstrated increased nuclear localization of *Nrf2* in comparison to normal and *Keap1* wild-type (Cal33 and *Keap1* wild-type patients’ tumor cells) cells (Fig. 3B). Since *Nrf2* controls the key components of the glutathione (GSH) and tightly regulates GSH levels by directly controlling glutamate-cysteine ligase complex (GCLC) and GCLM as well as glutathione S-transferase (GST) [28, 29] and thus cells acquire chemotherapeutic resistance, we investigated *Nrf2*-regulated target genes. Real-time RT-PCR analysis revealed that the majority of the *Nrf2*-regulated target genes were highly modulated in the cancer cells (Suppl. Fig. 3C). In addition, drug resistance markers MDR1 and ABCG2 were also highly upregulated in the cancer cells but not in the normal cells (Suppl. Fig. 3C). Importantly, as expected, the *Keap1* transcripts and proteins were downregulated in *Keap1* mutated cells (Suppl. Fig. 3C).

Several recent studies have reported that loss of *Keap1* alters cellular metabolic requirements and confers sensitivity to glutamine metabolism inhibitors [30–32]. These reports suggest that glutathione (GSH) production is increased by glutamine metabolism. In lung cancer cells, it was shown that glutaminase inhibition can sensitize the radiation-induced treatment resistance in the *Keap1* and *Nrf2* mutant cells [30]. We, therefore, aimed to determine whether loss of *Keap1* preferentially chemosensitizes by targeting glutaminase metabolism. First, we analyzed publicly available RNA-sequence data set GSE112026 and found significant overexpression of genes involved in glutamine metabolism (Fig. 3C). Next, we explored the possibility if targeting glutamine metabolism can chemosensitize *Keap1* mutant cells. We used a combination of chemotherapeutic agent cisplatin and a small-molecule glutamine inhibitor CB-839, which is currently under investigation in phase 1 and 2 clinical trials. Our results showed that although treatment of cells with CB-839 alone did not show significant sensitivity to CB-839 in *Keap1* mutant SSC9 and *Keap1* wild-type Cal33 cells. However, the combination of cisplatin and CB-839 significantly increased the sensitivity to combination treatment and killed a substantial number of cells in *Keap1* mutant SSC9 cells (Fig. 3D). In addition, the combination treatment significantly abolished the sphere growth efficiency in *Keap1* mutant SSC9 cells suggesting that the combination treatment may exhibit the potential to inhibit the self-renewal capacity of *Keap1* mutant cells (Fig. 3E). Furthermore, we noticed substantial inhibition of sphere growth effect in *Keap1* wild-type Cal33 cells after silencing by *Keap1*-siRNA (Fig. 3F). To identify by which mechanisms CB-839 preferentially chemosensitize the cells, we assessed the intracellular ROS levels after treatment with CB-839 and cisplatin. The baseline ROS levels in *Keap1* mutant SSC9 cells showed lower than in wild-type cells (Fig. 3G). However, unlike *Keap1* wild-type cells, a combination of cisplatin and CB-839 treatment significantly increased the ROS levels in *Keap1* mutant SSC9 cells compared to cisplatin alone treatment (Fig. 3G). Additionally, the CB-839 treatment significantly reduced the GSH activity in *Keap1* mutant SSC9 cells (Fig. 3H). These results suggest that CB-839 treatment preferentially follows the inhibition of free radical scavenging capacity in *Keap1* mutant cells compared with *Keap1* wild-type counterpart. We then tested the hypothesis that the addition of exogenous free radical scavenger preferentially rescues the capacity of *Keap1* mutant cells from CB-839-mediated chemosensitization. Our results showed that treatment of *Keap1* mutant SSC9 cells with a ROS scavenger NAC did not show significant effects on cell survival by NAC alone or either 10 μM cisplatin or CB-839 alone (Fig. 3I). Importantly, treatment of cells by NAC significantly rescued the increased cell death which was caused by the combination treatment of CB-839 and 10 μM of cisplatin (Fig. 3I).

### Loss of *Keap1* increases the *Nrf2* transcriptional activity, cancer stem cells characteristics in HNSCC

**Fig. 4:**
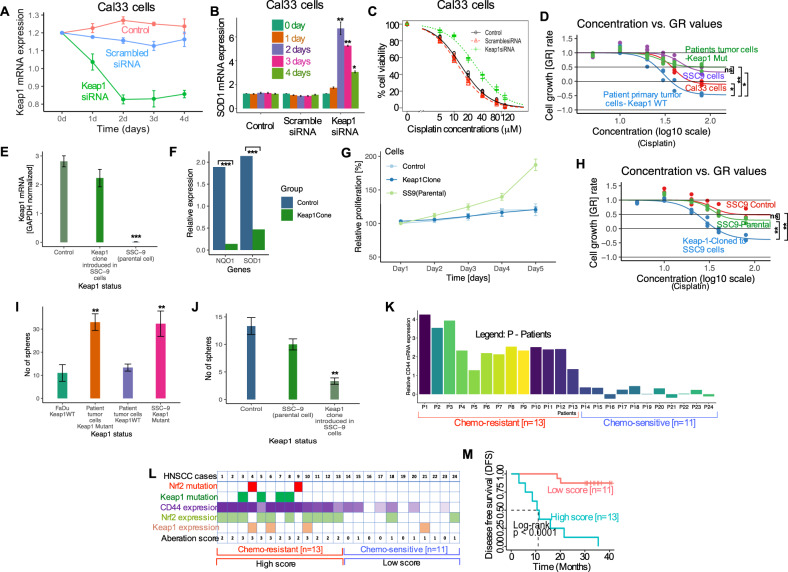
Loss of *Keap1* increases the *Nrf2* transcriptional activity, increase cancer stem cell characteristics, and predictor of chemotherapeutic outcome in patients with HNSCC. **A** Cal33 cells were transfected with siRNA against *Keap1*, scrambled, and control for 96 h. *Keap1* mRNA was assessed by quantitative RT-PCR. Results expressed as fold-change. **B** Cal33 cells were transfected as described in **A** and SOD1 mRNA was assessed by quantitative RT-PCR. **C** Cells were treated with *Keap1* siRNA to knock down the *Keap1* gene and assessed the cell viability 72 h after cisplatin treatment in the indicated concentrations in Cal33 cells. Data presented as mean SD of triplicate experiments. **D** Cell survival at 72 h after cisplatin treatment of indicated HNSCC patient’s primary tumor and HNSCC cell lines (*P < 0.05, **P < 0.01). **E** qRT-PCR analysis of *Keap1* expression in control, *Keap1* expressing SSC9 clone and parental SSC9 cells (***P < 0.001). **F** qRT-PCR analysis of *Nrf2* target genes SOD1 and NQO1 in control, *Keap1* expressing clone, and parental SSC9 cells (***P < 0.001). **G** Cell proliferation activity of *Keap1* expressing clone, control, and parental SSC9 cells. **H** Cell survival at 72 h after cisplatin treatment in parental SCC9, mock-transfected and *Keap1*-expressing clones. **I** Relative number of tumorspheres generated by the indicated patient’s tumor cells and cell lines. **J** Relative number of tumorspheres in parental SCC9, mock-transfected, and *Keap1*-expressing clone (**P < 0.01). **K** Expression of CD44 in cisplatin-resistant (*n* = 13) and cisplatin-sensitive (*n* = 11) HNSCC patients. **L** Summary of the results for the CD44 expression analysis in the presence of *Keap1* or *Nrf2* mutations and/or *Keap1* or *Nrf2* protein expression in each case (*n* = 24). The number of aberrations in each case was represented as the aberration scores (0, 1, 2, and 3) and all 24 cases were assigned into two groups based on the aberration scores: a “high score group” (*n* = 13 as aberration score 2 and 3) and low score group (*n* = 11 as aberration score 1, and 0). **M** Kaplan–Meier disease-free survival curve for 24 patients was generated according to the aberration score. The high score group was significantly associated with shorter disease-free survival (Log-rank *p* < 0.0001).

To get more insight into the *Keap1* mutations and resulting chemoresistance through *Nrf2* activation, we assessed the effects of siRNA knockdown of *Keap1* on the sensitivity of Cal33 tumor cells to cisplatin. *Keap1* siRNA was transfected into Cal33 cells under the treatment of 10 μM cisplatin. On days 1 and 2, siRNA against *Keap1* steadily reduced *Keap1* mRNA and maintained at this level for two days and again increased but stayed below baseline levels on days 3 and 4, while untreated control and scrambled-siRNA treated cells retained higher levels or on the baseline level (Fig. 4A). To identify if the knockdown of *Keap1* also activates the transcriptional activity of *Nrf2* target genes, we assessed SOD1 expression following *Keap1* siRNA knockdown. *Keap1* knockdown substantially increased the expression of SOD1 transcript by 6.2 and 5.1-fold on days 2 and 3 (Fig. 4B). In contrast, no significant change in SOD1 transcript was observed in control and scrambled siRNA-treated cells (Fig. 4B).

Next, we tested the cisplatin sensitivity in *Keap1* siRNA-transfected cells. Untreated control and scrambled-siRNA-treated cells showed sensitivity to cisplatin. Although, *Keap1* siRNA treated cells showed resistance at a lower dose but showed sensitivity to cisplatin at higher doses (Fig. 4C), and a higher EC50 value was recorded in *Keap1*-siRNA (the EC50 to cisplatin was 32.3 μM for *Keap1* siRNA-transfected cells and 7.2 μM and 8.7 μM for control and scrambled-siRNA, respectively. (*p* < 0.05 displays the differences between *Keap1* siRNA and control and scrambled groups).

Next, we sought to examine if the alterations of *Keap1* show any associations with chemotherapy resistance in HNSCC cells. To establish the functional differences in sensitivity to chemotherapy in *Keap1*-WT and *Keap1*-mutant cells, we examined whether loss of *Keap1* showed any effect on cell survival. Treatment of cells with cisplatin showed that *Keap1*-mutant SSC9 and patients’ primary tumor cells had resistance to cisplatin compared to *Keap1* wild-type cells (Fig. 4D). To explore whether the restoration of *Keap1* in *Keap1*-mutant cells affects cancer cell growth, we established a clone of SSC9 cells that stably express *Keap1* cDNA. The results showed the expression of *Keap1* mRNA in control and *Keap1* clone cells while the absence of *Keap1* mRNA in parental SSC9 cells (Fig. 4E). We further assessed changes in the expression of *Nrf2* target genes SOD1 and NQO1 in the *Keap1* clone cells. We found that restoration of *Keap1* expression eliminated the SOD1 and NQO1 gene expression (Fig. 4F). In addition, we compared the cell proliferation activity in the *Keap1-*expressing clone. We found that *Keap1* clone cells grew comparatively slower than the parental and mock-transfected control cells (Fig. 4G). Next, we examined the cisplatin sensitivity by reintroducing the *Keap1* clone in parental SSC9 cells. *Keap1*-expressing clone demonstrated poorer cell survival after cisplatin treatment for 72 h than parental and control cells (Fig. 4H). Furthermore, loss of *Keap1* showed extraordinary self-renewal capacity in *Keap1* mutant cells (Fig. 4I). Consistent with baseline differences in cell growth, *Keap1* expressing clones showed poorer tumorsphere formation after cisplatin treatment with clear contrast in parental and control cells (Fig. 4J), suggesting additional evidence of therapeutic resistance in HNSCC through self-renewal of the tumor cells.

Next, we examined the level of a well-known cancer stem cell (CSC) marker CD44 mRNA by qRT-PCR in chemoresistant (*n* = 13) and sensitive (*n* = 11) HNSCC patients’ samples. Twelve chemoresistant (54.17%) out of 24 patients treated with chemotherapy showed higher expression of CD44 (>50%) compared with the corresponding chemosensitive group (Fig. 4K). In addition, mutation analysis (review Figs. 1 and 2) led us to find *Keap1* mutations in four cases (17%) and Nrf2 in two cases (8%). On the basis of a positive aberration score for the mutations of *Keap1* and *Nrf2* and/or CD44 expression, we assigned 24 cases to two groups: 13 chemoresistant cases with 2–3 aberration scores to a “high group” and 11 chemosensitive cases with 0 and 1 aberration score to a “low score group” (Fig. 4L). Interestingly, the high score group (chemoresistant group) had worse DFS (Fig. 4M; Log-rank *P* < 0.0001). These results suggest that a fraction of patients treated with chemotherapeutic agents experience resistance to treatment, enhancing the CSC marker CD44 expression in addition to *Keap1* mutations and *Nrf2* activation in HNSCC and impacting the patients’ overall treatment outcome.

### Knockdown of *Nrf2* in *Keap1* defective cells leads to activation of ROS-mediated stress pathway and enhances the chemosensitivity

**Fig. 5:**
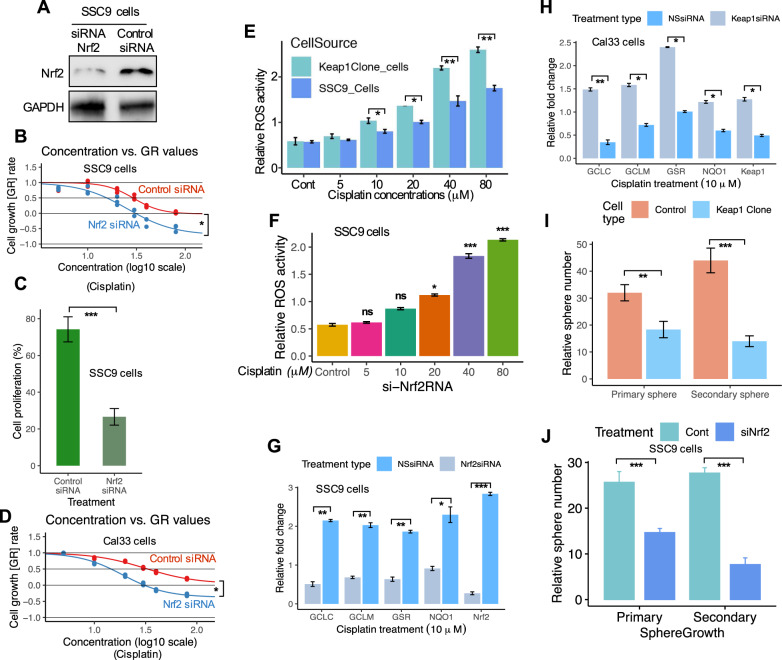
Knockdown of *Nrf2* in *Keap1* defective cells leads to activation of ROS-mediated stress pathway and enhances the chemosensitivity. **A** *Nrf2* expression in SSC9 cells transfected with control or *Nrf2*-siRNA. GAPDH was shown as a control. **B** Cell survival at 72 h after cisplatin treatment in control and *Nrf2-siRNA-treated* SSC9 cells. **C** Cell proliferation of SSC9 cells after treatment with control and *Nrf2*-siRNA. **D** Cell survival at 72 h after cisplatin treatment in control or *Nrf2*-siRNA-treated Cal33 cells. **E** Intracellular ROS level measured by DCFDA staining of SSC9 and *Keap1*-expressing clone cells. **F** Intracellular ROS level measured by DCFDA staining of SSC9 cells under the treatment of *Nrf2*-siRNA and cisplatin. **G** Silencing of *Nrf2* in cisplatin-treated SSC9 cells and analysis of *Nrf2-*dependent genes. **H** Inhibition of *Keap1* expression by *Keap1*-siRNA in Cal33 cells and analysis of *Nrf2*-dependent genes. **I** Analysis of a relative number of spheres generated in primary and secondary sphere cultures in SSC9 and *Keap1* clone cells. **J** Analysis of a relative number of spheres generated in primary and secondary sphere cultures in *Nrf2* knockdown SSC9 cells. Each experiment was repeated in triplicates. Data presents as mean ± SEM (*P < 0.05; **P < 0.01, ***P < 0.001).

Next, we assessed the role of *Nrf2* activation and chemoresistance in *Keap1*-mutant SSC9 cells. First, we silenced *Nrf2* expression by siRNA for *Nrf2* and assessed chemosensitivity. Silencing *Nrf2* by siRNA significantly reduced the endogenous *Nrf2* expression and activity in SSC9 cells (Fig. 5A). Cells treated with *Nrf2* siRNA showed increased sensitivity to cisplatin treatment in comparison with control siRNA-treated cells (Fig. 5B), concomitant with the decrease in cell proliferation in *Keap1*-mutant SSC9 cells (Fig. 5C; *p* < 0.001). In addition, we tested cisplatin sensitivity in Cal33 cells (*Keap1* WT) and observed high sensitivity to cisplatin in *Nrf2* knockdown cells (Fig. 5D; *p* < 0.05).

Cisplatin induces intrinsic apoptosis by producing mitochondrial ROS [33] leading to the induction of apoptosis. Furthermore, various antioxidant enzymes are induced by *Nrf2* activation and reduce the intracellular ROS level, therefore, resulting in the cells becoming more resistant to chemotherapies [19]. Moreover, *Nrf2* directly affects the homeostasis of ROS by regulating the antioxidant defense system [34]. *Nrf2*-mediated chemotherapeutic resistance is likely to occur due to the reduction of drug-induced ROS. Considering these, we hypothesized that *Nrf2*-induced chemotherapy resistance might be partially due to the decrease in drug-induced ROS generation. To address this question, we treated mock and *Keap1*-expressing SSC9 clones with cisplatin and assessed the mitochondrial ROS production using a fluorescent indicator. At the same time, we analyzed the ROS level in *Nrf2*-siRNA-treated SSC9 cells. We observed that both the *Keap1*-expressing clone and *Nrf2* knockdown cells showed higher ROS generation under the treatment of cisplatin (Fig. 5E, F), suggesting that increased proliferation (Fig. 5C) was seen in *Keap1*-mutated cells is largely mediated by *Nrf2*. To further demonstrate that *Nrf2* activation contributes to the increased expression of antioxidants, and xenobiotic metabolism enzymes, we challenged cells with *Nrf2* siRNA in SSC9 cells. Transfection of *Nrf2*siRNA in cells decreased the *Nrf2* mRNA by 70–75% with the reduction of *Nrf2* target genes (Fig. 5G). Conversely, inhibition of *Keap1* expression by siRNA increased the *Nrf2* target genes in Cal33 cells (Fig. 5H). In addition, in vitro sphere formation assay revealed that *Keap1*-expressing cells decreased the growth of spheres by 1.5-fold compared with parental SSC9 cells under the cisplatin treatment condition (Fig. 5I). The growth of the sphere continued to decrease further in the secondary sphere culture approximately by 1.5–2.0-fold (Fig. 5I). In addition, *Nrf2* knockdown impaired tumorsphere growth in these cells confirmed that overexpression of *Nrf2* contributes to a stem-like phenotype in HNSCC cells (Fig. 5J). These data indicate that loss of *Keap1* in HNSCCs leads to increased cell proliferation, and expression of *Nrf2*, as well as sphere growth efficiency, suggesting that *Nrf2* activation and decrease of ROS and chemotherapeutic resistance is the vital mechanistic mediator observed in loss of *Keap1*.

### *Keap1* mutations and *Nrf2* overexpression regulates Notch signaling in HNSCC cells

**Fig. 6:**
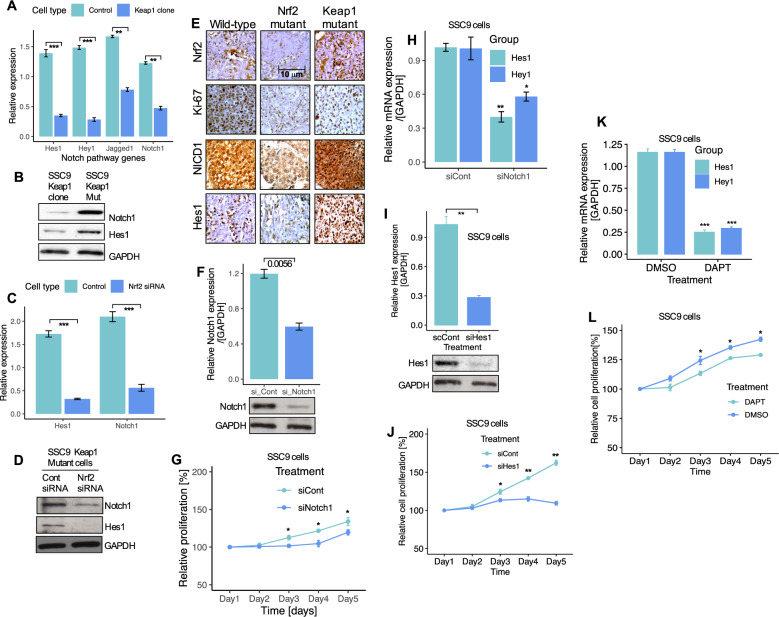
Nrf2 regulates Notch signaling in HNSCC cells. **A** Expression of Notch1 and Notch target genes mRNA in control and *Keap1-*expressing SSC9 clone cells. **B** Expression of Notch1 and Hes1 proteins in *Keap1*-mutant and *Keap1-*expressing SSC9 cells. **C** Notch1 and Hes1 mRNA and, **D** protein expression after Nrf2 knockdown in SSC9 cells. **E** Immunohistochemistry staining and expression of *Nrf2*, Ki67, Notch1, and Hes1 in HNSCC clinical samples from wild-type, *Nrf2*, and *Keap1* mutant patients tumor tissues. **F** Notch1 expression in non-targeting control and Notch1 siRNA-treated SSC9 cells **G** Cell proliferation of SSC9 cells after knockdown of Notch1 by siRNA. **H** Relative mRNA expression of Hes1 and Hey1 after Notch1 knockdown in SSC9 cells. **I** Hes1 mRNA expression and, **J** Cell proliferation after knockdown of Hes1 siRNA in SSC9 cells. **K** Effects of Notch inhibitor DAPT and, **L** Assessment of cell growth after treating the cells with Notch inhibitor DAPT for 5-days. The mRNA expression levels were calculated and normalized relative to GAPDH. All experiments were run in triplicate and compared with the control group. Data presents as mean ± SEM (**P < 0.01, ***P < 0.001).

Our clinical results indicate that *Keap1* mutations are strongly associated with chemo-radio resistance. However, patients with *Keap1* mutations developed tumor regrowth in the lung. Furthermore, loss of *Keap1* and *Nrf2* activation has previously been reported to confer resistance to chemotherapy [35, 36]. Recent studies have reported that the Notch target genes show direct downstream transcriptional mediators of *Nrf2* signaling [37–40]. Moreover, previous studies have reported that activation of the Notch signaling enhances self-renewal of oral squamous cell carcinoma cancer cells, while its loss impairs the maintenance of self-renewal [41]. Therefore, we hypothesized that the *Keap1*-*Nrf2* pathway likely modulates activation of the Notch pathway. To explore this, we first measured the expression of Notch1 and Notch target genes in *Keap1-*expressing clone cells. Notch1 and Notch target genes prominently decreased in *Keap1*-expressing cells (Fig. 6A), while their expression significantly increased in *Keap1* mutant cells. Next, we tested if the *Nrf2* overexpression and *Keap1* mutations may have any effects on Notch activity. The expression levels of Notch1 and Hes1 were decreased in *Keap1* expressing cells compared to *Keap1* mutant cells (Fig. 6B). On the other hand, *Nrf2* knockdown cells expressed significantly lower levels of Notch1 and Hes1 mRNA (Fig. 6C), and protein compared to controls (Fig. 6C, D). These results suggest that *Keap1*-*Nrf2* regulates Notch signaling in HNSCC. We obtained tumor tissues from *Keap1* mutant, wild-type, and *Nrf2* mutant patients’ samples and immunostained them for the expression of Notch1, Hes1, Ki67, and *Nrf2*. Immunostaining results confirmed the absence of *Nrf2* in *Nrf2* mutant tumor tissues and high levels of *Keap1* mutant tumors (Fig. 6E). Ki-67, a cell proliferation marker, was highly expressed in *Keap1*-mutant tumors compared with those in *Nrf2* mutant tumors (Fig. 6E). Expression of Notch1 and Hes1 were significantly highly expressed in *Keap1* mutant tumors as compared with those of *Nrf2*-mutant tumors (Fig. 6E). These results indicated the functional role of the *Keap1*-*Nrf2* pathway in regulating the cell proliferation and active involvement of the Notch signaling HNSCC tumors.

As shown in Figs. 5J and 6D, knockdown of *Nrf2* significantly impaired tumorspheres and downregulation of Notch1 and Hes1. Therefore, we tested the possibility of the Notch pathway as a target for directed therapy by exploring its functional consequences in *Nrf2*-activated HNSCC cells with *Keap1* mutations. First, we inhibited Notch1 activity by siRNA in SSC9 cells. Following transfection of cells with Notch1 siRNA, cells showed a significant decrease in cell proliferation (Fig. 6F, G), coupled with a significant reduction in two Notch pathway target genes, Hes1 and Hey1 (Fig. 6H). We then assessed whether inhibition of Hes1 also shows any impact on cell growth. Congruent with the results obtained for Notch1 inhibition in Fig. 6G, inhibition of Hes1 significantly decreased cell proliferation (Fig. 6I, J). Similarly, treatment of cells with a Notch pathway inhibitor DAPT significantly inhibited cell growth (Fig. 6K, L).

### *Keap1* mutation is a strong predictor of chemotherapeutic outcomes in patients with advanced HNSCC

**Fig. 7:**
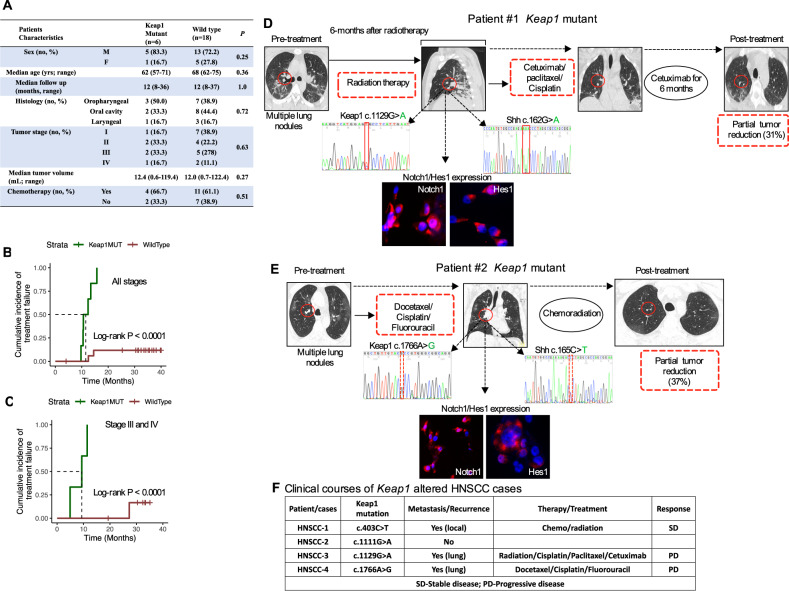
Combination therapy with cetuximab, paclitaxel, and cisplatin led to a partial response in a patient with *Keap1* mutant advanced-stage metastatic HNSCC. **A** Clinical characteristic of head and neck cancer patient cohort treated with chemotherapy and analyzed for *Keap1* and *Nrf2* mutation by Sanger sequencing. **B**, **C** Association *Keap1* mutations and local treatment failure in patients with HNSCC treated with chemotherapy. **B** Patient cohort and, **C** Stage III–IV patients who were treated with chemotherapy. **D**, **E** Tumor progression in an index patient with lung metastasis was associated with the identification of *Keap1* and Shh mutations and tumors from index patients with *Keap1* mutant strongly expressed Notch1 and Hes1. In both cases, patients with *Keap1* mutations achieved a partial response to 31% and 37% reduction, respectively, in the metastatic lung region upon treatment with two lines of chemoradiation/cetuximab (patient case #1) and three cycles of TPE (docetaxel, cisplatin, and fluorouracil (TPF) followed by chemoradiation with cisplatin treatment (patient case #2). **F** Clinical courses/outcomes of *Keap1* mutant HNSCC patient treated with chemoradiation therapy. SD stable disease, PD progressive disease.

Given the fact that *Keap1* mutations and *Nrf2* overexpression lead to chemotherapeutic resistance, we, therefore, hypothesized that *Keap1* mutations might lead to an increased rate of local recurrence in advanced HNSCC patients treated with chemotherapeutic agents. As described in Figs. 1 and 2, patients with *Keap1* mutations were predicted to be deleterious and had a cumulative incidence of local treatment failure at ten months which was 80% in patients whose tumors carried *Keap1* mutations as compared with <12% in patients with wild-type tumors (Log-rank *p* < 0.0001), while analyzing the patients with higher stages, particularly in patients with stage III–IV had higher rates of treatment failure in patients who harbored *Keap1* mutations (Log-rank *p* < 0.0001) (Fig. 7A–C).

### Combination therapy with cetuximab, paclitaxel, and cisplatin led to a partial response in a patient with *Keap1* mutant advanced-stage metastatic HNSCC

The *Keap1-Nrf2* pathway has been shown to cancer cell survival and mutations in *Keap1* or *Nrf2* are clinically relevant predictive biomarkers of chemo-radio resistance [30]. The standard chemotherapy for advanced-stage HNSCC patients are cisplatin, 5-fluorouracil, and docetaxel/paclitaxel, and show improved progression-free and overall survival [42]. Furthermore, the most common sites of distant metastases were reported to be the lung (70%) followed, by the liver (42%) and bones (15%) [43, 44]. However, chemotherapy resistance results in poor treatment outcomes, and the reasons for chemotherapy resistance are diverse and multifaceted. To identify the *Keap1* mutations and associated chemo-radio resistance, we present two case reports that recently underwent a combination of chemotherapy and related resistance to therapy. Patient #1 is a 59-year-old male visiting the clinic with advanced metastatic squamous cell carcinoma of the laryngeal. The patient received initial radiotherapy followed by two lines of chemotherapy (cetuximab/paclitaxel/cisplatin [CPP]). The patient had rapid disease progression and underwent biopsy and genotyping of lung metastasis that revealed *Keap1* mutations (Fig. 7D). Following CPP, the patient was then treated with cetuximab weekly for six months and achieved a partial response (PR) but had rapid disease progression and eventually succumbed as a consequence of the disease. Patient #2 is a 64-year-old male diagnosed with metastatic oral cavity cancer and initially treated with laryngeal-preservation surgery, followed by three cycles of docetaxel, cisplatin, and continuous infusion of fluorouracil followed by a chemoradiation with cisplatin. The patient achieved a PR. Approximately after 9 months, recurrence was observed, and CPP was initiated. Following CPP, the patients had rapid disease progression and underwent biopsy and genotyping of lung metastasis and found *Keap1* mutations (Fig. 7E). Further disease progression of oral cavity cancer was observed shortly after the completion of cycle 3. In both cases, sequencing of cells from lung metastatic site showed *Keap1* and Shh mutations and strong expression of Notch1 and Hes1 confirmed the activation of the Notch pathway. Thus, among recurrent and metastatic HNSCC patients, *Keap1* mutations appear to be the most significant cause of clinical chemo-radio resistance (Fig. 7F) and are coupled with the activation of the Notch signaling pathway.

## Discussion

Loss of *Keap1* enhances the nuclear accumulation of *Nrf2* followed by the elevated expression of anti-oxidative, anti-xenobiotic stress enzymes and drug efflux pumps [45]. In the present study, we sought to elucidate the alterations of the *Keap1*-*Nrf2* pathway and identify the mechanisms of chemotherapeutic resistance in HNSCC. We identified mutations that occur more frequently in the oral cavity in HNSCC, associated with the changes in amino acids in 4 (17%) tumors out of 24 HNSCC patient tumors sequenced, which is much higher than the TCGA data set. The possible putative reason for the higher incidence of *Keap1* mutations in our case is unknown however, we speculate that demographic and genetic makeup may play roles in the higher incidence of *Keap1* mutations in HNSCC patients. In this study, we found that all *Keap*1 mutated tumors exhibited nuclear accumulation of *Nrf2* as assessed by immunohistochemical analysis. *Keap1* mutations result in the accumulation and activation of *Nrf2* and may partly confer the resistance to chemotherapeutic treatment in HNSCC patients and reduce drug-induced ROS production. Importantly, all these mutations involved functionally relevant domains of *Keap1* protein, including BTB (c.403 C > T and c.1129 G > A), IVR domain (c.1111 G > A), and KR3 region (c.1766A > G) of *Keap1*, which are responsible for the ubiquitination and binding of *Nrf2* [46, 47]. The activation of the *Nrf2* pathway has been proposed to be the leading cause of chemoresistance in several cancers [48, 49]. To identify the mechanisms associated with *Nrf2* pathway activation in HNSCC, we sequenced the *Keap1* and *Nrf2* genes and identified mutations in *Nrf2* immunopositive tumors. Interestingly, the *Nrf2* mutations involved the Neh2 domain where *Keap1* binds. Importantly, we did not detect any mutations in the matched normal tissues, confirming that the mutation is somatic in origin. Furthermore, the overall frequency of mutations (17%; 4/24) for the *Keap1* gene in HNSCC tissues suggests that *Keap1* mutations are likely a frequent genetic alteration in HNSCC at least in our case which is much higher than that of data reported in TCGA. In addition, we assessed whether the *Nrf2* activation pathway is engaged in mediating chemotherapeutic resistance in HNSCC. We used human HNSCC primary tumor cells and HNSCC cell lines to evaluate the functional relationship between *Nrf2* activation and chemotherapeutic resistance. Knockdown of *Keap1* by siRNA in HNSCC cells demonstrated enhanced *Nrf2* pathway activity, which led to enhanced transcriptional activity thereby rendering HNSCC cells resistant to chemotherapy. We have achieved a comparable result in HNSCC tumor and normal tissue, where the loss of functional *Keap1* gene and subsequently increased staining intensity of *Nrf2* corroborate the above findings. In concordance with the above findings, as expected, GST, NQO1, and SOD1 enzyme levels and GSH levels were highly significantly elevated in the tumor tissues compared with matched normal individuals. This suggests that upregulation of these *Nrf2*-dependent genes likely contributed to the resistance to chemotherapy treatment and cell survival. This result is in agreement with previously published results, where high antioxidant capacity increases cell survival and proliferation and protects against oxidants, radiation therapy, and chemotherapies [10].

Loss of *Keap1* and *Nrf2* overexpression induces many stress resistance genes and can restore cancer cell proliferation. It has previously shown that constitutive activation of *Nrf2* contributes to tumorigenesis, ROS detoxification, and modulation of redox state and also contributes to resistance to many anticancer drugs [35, 50]. Loss of *Keap1* has been identified as a possible mechanism of chemoradiation resistance in many cancers [51]. In the lung cancer cells, *Keap1* mutations showed more resistance to etoposide and carboplatin than to *Keap1* wild-type cells [45]. Thus, overexpression of *Nrf2* due to *Keap1* loss may confer resistance to cisplatin, a widely used chemotherapy regimen for head and neck cancer, by regulating ROS and cancer stem cell pathways. In concordance with these previous results, our results demonstrated that downregulation of the *Nrf2* expression in *Keap1* mutated cancer cells or introduction of *Keap1* cDNA in *Keap1* mutant cells significantly enhances the sensitivity to cisplatin. In analyzing the clinical cases, all four *Keap1* mutated HSNCC patients had a history of recurrence within a year all cases were treated with 2 and 3 lines of chemotherapy and exhibited poor clinical response, suggesting therapeutic resistance due to *Keap1* mutations as well as the potential existence of CSC populations in these tumors.

Our observation from this study suggests a pivotal role for *Keap1* mutations during HNSCC oncogenesis due to the deletion of *Keap1* led to the increased self-renewal activity of cancer cells and subsequent therapeutic resistance. Importantly the impact of *Keap1* loss leads to increased self-renewal activity and therapeutic resistance compared to *Keap1* wild-type cells or *Keap1* reintroduced clones which define the greater clinical relevance. Previous reports have demonstrated the molecular mechanisms of cisplatin resistance in cancer cells and cisplatin treatment increased mitochondrial ROS generation [52] and triggered the apoptosis process [53, 54]. In our analysis, when we restored *Keap1* or silenced *Nrf2* observed increased mitochondrial ROS generation and limited the cell growth in cells treated with cisplatin. This suggests that constitutive *Nrf2* activation due to *Keap1* loss influences oxidative stress and reduces the cellular damage induced by the increase of ROS, therefore, triggering the resistance of chemotherapy, particularly for cisplatin. Han and colleagues [41] recently reported the interaction of *Nrf2* and Notch signaling in OCSC (oral squamous cell carcinoma) cells. Activation of *Nrf2* in *Keap1* deleted cells resulted in hyperproliferation of squamous epithelial cells and activation of Notch signaling [41]. Congruent with this finding, our results report a role in chemoresistance on HNSCC cells which is mediated through increased Notch signaling and increased Notch pathway components upon *Nrf2* activation in *Keap1* mutated cells, suggesting that Notch also plays a role in mediating the effects of *Nrf2* activation in HNSCC cells.

Reviewing all our results, it appears that once HNSCC cells acquire a mutation in *Keap1*, activation of *Nrf2* and the Notch signaling pathway promotes cellular metabolic reprogramming that sustains cellular proliferation. This reprogramming feature leads to the clonal expansion of mutant cells thus acquiring self-renewal capacity and triggering resistance pathways and outpacing the *Keap1* WT cells and potentially acquiring additional genetic changes and subsequently leading to therapeutic failures. All these features explain the therapeutic failure and adverse and poor survival outcomes conferred by *Keap1* mutations in rapidly progressing HNSCC cells. In a recent study, it was reported the *Keap1*-*Nrf2* double mutations in lung cancer leads to poor outcome and severe therapeutic resistance in radiation therapy [38]. In our study, we found two patients with *Nrf2* mutation and none of the patients had concurrent *Keap1-Nrf2* mutations. This may be due to the small number of patients assessed in this study and we cannot rule out that *Keap1-Nrf2* dual mutations may have somatic or epigenetic consequences and may play a devastating role in therapeutic failures and poor outcomes, disease recurrence in *Keap1-Nrf2*-mutant tumors. Thus, future studies should include large numbers of patients’ samples to examine the effects of *Keap1* mutation as well as to explore the role of *Keap1-Nrf2*-dual mutation in chemoresistance.

In conclusion, although *Keap1*–*Nrf2* alterations are known to play roles in chemotherapeutic resistance particularly cisplatin resistance in HNSCC, surprisingly, the mutation status is not widely used to make a treatment decision in head and cancer. It would be interesting to investigate whether loss of *Keap1* and overexpression of *Nrf2* status in tumor samples are clinically relevant mechanisms of chemotherapeutic resistance in HNSCC and develop alternative therapy to counter *Nrf2* activation. This may improve personalized therapy in a subset of HNSCC patients who are prone to therapeutic resistance.

## Supplementary information

Supplementary Figure Legends

Supplementary Figure S1

Supplementary Figure S2

Supplementary Figure S3

Supplementary Table S1

Supplementary Table S2

Supplementary Table S3

Supplementary Table S4

Supplementary Table S5

Supplementary Table S6

Supplementary Table S7

Reporting summer/Reproducibility checklist

Original Data File

## Data Availability

The data that support our results in this follow-up study are available from the corresponding authors upon reasonable request with prior permission from Parkview hospital, Chittagong, Bangladesh.
