# Supplementary material for: Genetic alterations of Keap1 confers chemotherapeutic resistance through functional activation of Nrf2 and Notch pathway in head and neck squamous cell carcinoma
Source: Cell Death Dis. 2022 Aug 9;13(8):696. doi: 10.1038/s41419-022-05126-8 (PMC9363464; doi:10.1038/s41419-022-05126-8)
Supplement: Supplementary file 1 — Supplementary Figure Legends [file 41419_2022_5126_MOESM1_ESM.docx]

**Supplementary Figure legends**

**Supplementary Figure S1.** Distribution and alteration of *Keap1* and *Nrf2* in TCGA datasets. **A** Distribution analysis and **B-C** Position of *Keap1* and *Nrf2* mutations in fully characterized HNSCC samples from the TCGA database.

**Supplementary Figure S2.** Kaplan-Meier disease-free survival analysis curve of *Nrf2* mutant, *Keap1* mutant, and wild-type HNSCC patients (n=24, 2-*Nrf2* mutant, 4-*Keap1* mutant, and 18 wild-type patients) (Log-rank p<0.0001).

**Supplementary Figure S3.** Biological effects of *Keap1* mutations and *Nrf2* overexpression in HNSCC tumors and normal tissues. **A** Total GSH and enzyme activity analysis of SOD1, NQO1, and GST in HNSCC tumor cells and matched normal tissues. A red * indicates a tumor with *Keap1* mutations and a green ** star indicates a tumor with *Nrf2* mutations. **B** Immunofluorescence analysis of endogenous *Nrf2* protein in SSC9 cells. Arrow indicates the granular nuclear localization in the cells. **C** Heat map showing the relative expression of *Nrf2*, *Keap1,* and *Nrf2*-dependent genes and drug-resistance markers obtained by quantitative RT-PCR from indicated tissues and cell lines. Color key: 1 is the lowest expression and 4 is the highest possible expression. Raw data for the heatmap generated from qRT-PCR are presented in Supplementary Table S5.
