# Supplementary figures and images for: Genetic alterations of Keap1 confers chemotherapeutic resistance through functional activation of Nrf2 and Notch pathway in head and neck squamous cell carcinoma
Source: Cell Death Dis. 2022 Aug 9;13(8):696. doi: 10.1038/s41419-022-05126-8 (PMC9363464; doi:10.1038/s41419-022-05126-8)

# Supplementary Figure S1

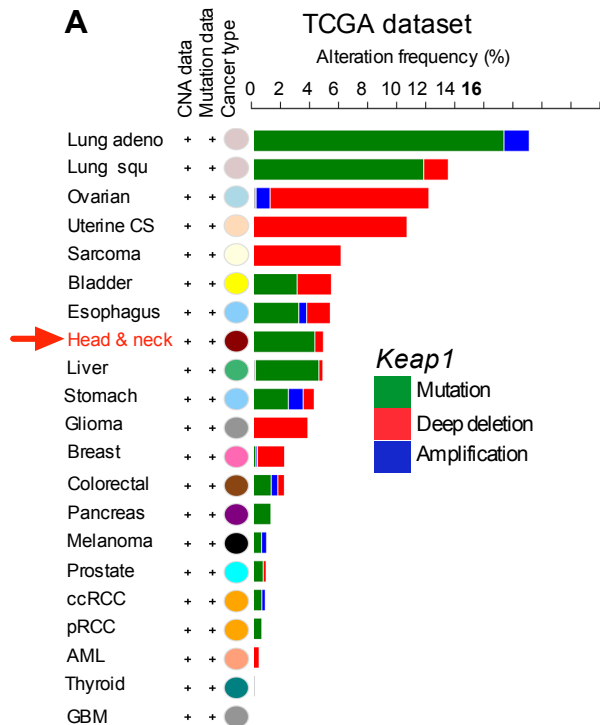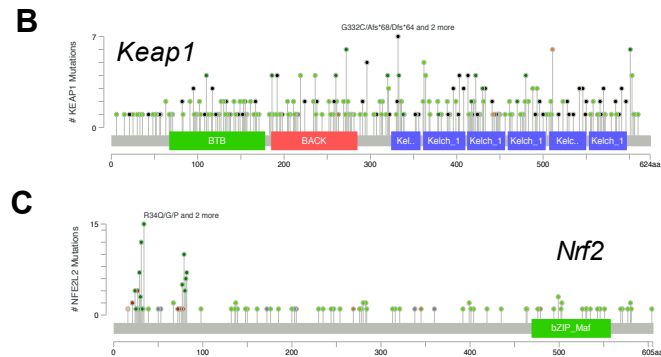

Supplement: Supplementary file 2 — Supplementary Figure S1 [file 41419_2022_5126_MOESM2_ESM.pdf]

# Supplementary Figure S2

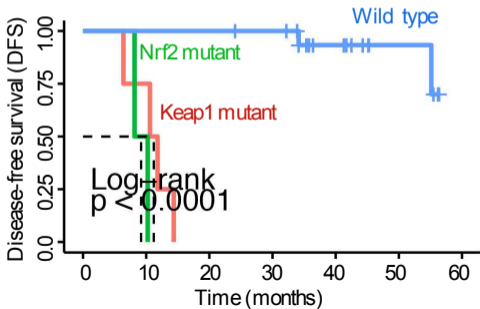

Supplement: Supplementary file 3 — Supplementary Figure S2 [file 41419_2022_5126_MOESM3_ESM.pdf]

Supplementary Figure S3

**A**

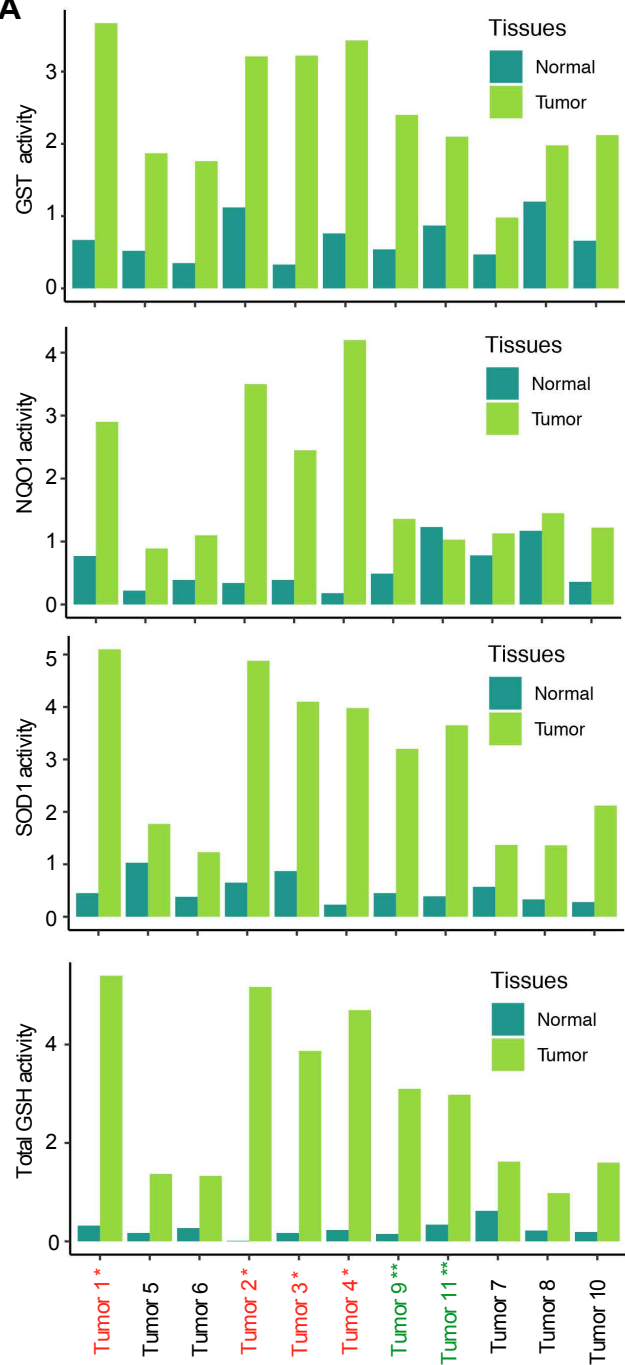

**B**

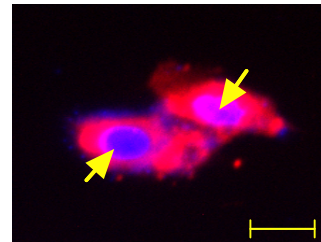

**C**

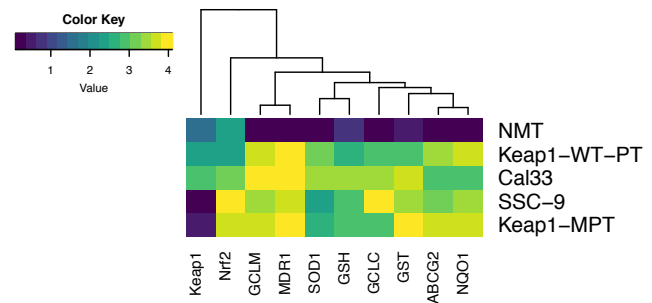

Supplement: Supplementary file 4 — Supplementary Figure S3 [file 41419_2022_5126_MOESM4_ESM.pdf]
