# Supplementary material for: Genetic alterations of Keap1 confers chemotherapeutic resistance through functional activation of Nrf2 and Notch pathway in head and neck squamous cell carcinoma
Source: Cell Death Dis. 2022 Aug 9;13(8):696. doi: 10.1038/s41419-022-05126-8 (PMC9363464; doi:10.1038/s41419-022-05126-8)
Supplement: Supplementary file 5 — Supplementary Table S1 [file 41419_2022_5126_MOESM5_ESM.docx]

| Supplementary Table S1. Primers used in PCR amplifications from FFPE tumor sections for Sanger sequencing. | | | | |
| --- | --- | --- | --- | --- |
| **Primer** | **Gene** | **Coding Exon** | **Sequence** | **Amplicon size (bp)** |
| KEAP1Ex1F | KEAP1 | 1 | cctcatccagccctgtcttc | 303 |
| KEAP1Ex1R |  |  | ctcgatcacgtagaagacctt |  |
| KEAP1Ex2-2F | KEAP1 | 2 | caaggactacctggtcaagatc | 302 |
| KEAP1Ex2-2R |  |  | ggttgtaacagtccagggc |  |
| KEAP1Ex3F | KEAP1 | 3 | tccacgaaggtcagctataatgg | 254 |
| KEAP1Ex3R |  |  | gcttcggatggtgttcattgc |  |
| KEAP1Ex4F | KEAP1 | 4 | gagtcaccttctctgcatgg | 248 |
| KEAP1Ex4R |  |  | gatgggctagtcaggactctt |  |
| KEAP1Ex5F | KEAP1 | 5 | gctgcatctctctctttctgtc | 239 |
| KEAP1Ex5R |  |  | gtacagttctgctggtcaatc |  |
| NRF2Ex2F | NRF2 | 2 | accatcaacagtggcataatgtg | 397 |
| NRF2Ex2R |  |  | ctgccataactttcccaagaac |  |
