# Supplementary material for: Genetic alterations of Keap1 confers chemotherapeutic resistance through functional activation of Nrf2 and Notch pathway in head and neck squamous cell carcinoma
Source: Cell Death Dis. 2022 Aug 9;13(8):696. doi: 10.1038/s41419-022-05126-8 (PMC9363464; doi:10.1038/s41419-022-05126-8)
Supplement: Supplementary file 6 — Supplementary Table S2 [file 41419_2022_5126_MOESM6_ESM.docx]

| Supplementary Table S2. List of primers for qRT-PCR | | |
| --- | --- | --- |
| Gene name | Forward primers | Reverse primers |
| ***Nrf2*** | GCGACGGAAAGAGTATGAGC | GTTGGCAGATCCACTGGTTT |
| ***Keap1*** | CCTTCAGCTACACCCTGGAG | CATGACCTTGGGGTGGATAC |
| ***GCLC*** | AGAGAAGGGGGAAAGGACAA | GTGAACCCAGGACAGCCTAA |
| ***SOD2*** | GCCATTGCTTTTGGTGTTTT | AAATGGTGCTGGGAAAACTG |
| ***GCLM*** | TGGAAATGCCCAACATTTTT | TGAGGGTGCAGGTAGGAGAT |
| ***NQO1*** | TTACTATGGGATGGGGTCCA | TCTCCCATTTTTCAGGCAAC |
| ***GSR*** | AGTGTTGACACTTACCGCCA | CCCTCAGGACTGATTTACTGCT |
| ***GSH*** | CCCCAACCTACTAGTTCGCC | CTCTTTAAGCCTAGCCGGGG |
| ***GST*** | CTCAAAAGGCTTCAGTTGCC | ACCTCCGCTGCAAATACATC |
| ***MDR1*** | GACCGGACATCCCAGTGCTT | TGTGCTCGGAGACACTGAAC |
| ***ABCG2*** | TGGCTGTCATGGCTTCAGTA | GCCACGTGATTCTTCCAC AA |
| ***Notch1*** | CCAGCATCACCTGCCTGTTA | CCAAGTCTGACGTCCCTCAC |
| ***Jagged1*** | GGCCGAGGTCCTATACGTTG | ACACAAGGTTTGGCCTCACA |
| ***Hes1*** | ATGACAGTGAAGCACCTCCG | AAACACCTTAGCCGCCTCTC |
| ***Hey1*** | GTGCGGACGAGAATGGAAAC | TTGCTCCATTACCTGCTTCTCA |
