# Supplementary material for: Genetic alterations of Keap1 confers chemotherapeutic resistance through functional activation of Nrf2 and Notch pathway in head and neck squamous cell carcinoma
Source: Cell Death Dis. 2022 Aug 9;13(8):696. doi: 10.1038/s41419-022-05126-8 (PMC9363464; doi:10.1038/s41419-022-05126-8)
Supplement: Supplementary file 7 — Supplementary Table S3 [file 41419_2022_5126_MOESM7_ESM.docx]

| Supplementary Table S3: Missense mutations identified in head and neck cancer patients (n=24) | | | | | | | | |
| --- | --- | --- | --- | --- | --- | --- | --- | --- |
|  |  |  |  |  |  | In silico predictors | | |
| Genes | Mutations | Proteins | Mutation types | dbSNP/COSMIC | Frequency | FATHMM^1^ | Polyphen^2^ | Mutation tester^3^ |
| Keap1 | c.403C>T | p.R135C | Somatic | COSM4433833 | 4.1 | Pathogenic (0.89) | Probably Damaging (1.0) | Disease causing (180) |
| Keap1 | c.1111G>A | p.G371S | Germ line | rs760899478 | 4.1 | NA | Probably Benign (0.0) | Disease causing (56) |
| Keap1 | c.1129G>A | p.V377M | Somatic | Novel | 4.1 | NA | Probably Damaging (0.97) | Disease causing (21) |
| Keap1 | c.1766A>G | p.D589G | Germ line | Novel | 4.1 | NA | Probably Damaging (1.0) | Disease causing (94) |
| Nrf2 | c.145G>A | p.E49K | Somatic | Novel | 4.1 | NA | Probably Damaging (1.0) | Disease causing (56) |
| Nrf2 | c.241G>C | p.G81R | Somatic | COSM5437128 | 4.1 | Pathogenic (1.0) | Probably Damaging (1.0) | Disease causing (125) |
| Notes: ^1^Functional Analysis through Hidden Markov Models, FATHMM v2.3 (0.0 to 1.0); ^2^PolyPhen-2 v2.2.2r398 (0.0 to 1.0); ^3^MutationTaster (0.0 to 215) | | | | | | | | |
