# Supplementary material for: Genetic alterations of Keap1 confers chemotherapeutic resistance through functional activation of Nrf2 and Notch pathway in head and neck squamous cell carcinoma
Source: Cell Death Dis. 2022 Aug 9;13(8):696. doi: 10.1038/s41419-022-05126-8 (PMC9363464; doi:10.1038/s41419-022-05126-8)
Supplement: Supplementary file 8 — Supplementary Table S4 [file 41419_2022_5126_MOESM8_ESM.docx]

| Supplementary Table S4. Synonymous variants identified from tumors of head and neck cancer patients (n=24). | | | | | | | |
| --- | --- | --- | --- | --- | --- | --- | --- |
| **Genes** | **Variants** | **Proteins** | **Types** | **dbSNP** | **Frequency (%)** | **Global allele frequencies** | **Comments** |
| *KEAP1* | c.474T>C (A>G) | p.Gly158Gly | Germ line | rs1048287 | 4.1 | A=0.91980 G=0.08020 | *Lower in cancer population* |
| *KEAP1* | c.1152C>T (G>A) | p.Pro384Pro | Germ line | rs201119783 | 4.1 | G=0.99970 A=0.00030 | *Enriched in cancer population* |
| *KEAP1* | c.1413C>G (G>C) | p.Leu471Leu | Germ line | rs1048290 | 4.6 | G=0.58821 C=0.41179 | *No change* |
| *KEAP1* | c.1611C>T (G>A) | p.Tyr537Tyr | Germ line | rs11545829 | 8.2 | G=0.95921 A=0.04079 | *Enriched in cancer population* |
| *KEAP1* | c.1815C>T (G>A) | p.Gly605Gly | Gem line | rs139000421 | 4.1 | G=0.99997 A=0.00003 | *Enriched in cancer population* |
