# Supplementary material for: Genetic alterations of Keap1 confers chemotherapeutic resistance through functional activation of Nrf2 and Notch pathway in head and neck squamous cell carcinoma
Source: Cell Death Dis. 2022 Aug 9;13(8):696. doi: 10.1038/s41419-022-05126-8 (PMC9363464; doi:10.1038/s41419-022-05126-8)
Supplement: Supplementary file 9 — Supplementary Table S5 [file 41419_2022_5126_MOESM9_ESM.docx]

| Supplementary Table S5. Molecular profile of Keap1, Nrf2 and TP53 in primary head and neck cancer tumors | | | | |
| --- | --- | --- | --- | --- |
| **Cases** | **Mutation type** | **Keap1 mutation** | **Nrf2-IHC** | **TP53-IHC** |
| HNSCC-1 | Somatic | c.403C>T | 1+ | 2+ |
| HNSCC-2 | Germ line | c.1129G>A | 1+ | 2+ |
| HNSCC-3 | Somatic | c.1111G>A | 1+ | 2+ |
| HNSCC-4 | Germ line | c.1766A>G | 1+ | 2+ |
| HNSCC-5 |  | Wild-type | 0 | 2+ |
| HNSCC-6 |  | Wild-type | 0 | 1+ |
| HNSCC-7 |  | Wild-type | 0 | 0 |
| HNSCC-8 |  | Wild-type | 2+ | 0 |
| HNSCC-9 |  | Wild-type | 0 | 0 |
| HNSCC-10 |  | Wild-type | 0 | 2+ |
| HNSCC-11 |  | Wild-type | 1+ | 1+ |
| HNSCC-12 |  | Wild-type | 0 | 0 |
| HNSCC-13 |  | Wild-type | 0 | 1+ |
| HNSCC-14 |  | Wild-type | 0 | 0 |
| HNSCC-15 |  | Wild-type | 0 | 0 |
| HNSCC-16 |  | Wild-type | 2+ | 0 |
| HNSCC-17 |  | Wild-type | 0 | 2+ |
| HNSCC-18 |  | Wild-type | 0 | 0 |
| HNSCC-19 |  | Wild-type | 0 | 0 |
| HNSCC-20 |  | Wild-type | 0 | 1+ |
| HNSCC-21 |  | Wild-type | 1+ | 2+ |
| HNSCC-22 |  | Wild-type | 0 | 0 |
| HNSCC-23 |  | Wild-type | 0 | 0 |
| HNSCC-24 |  | Wild-type | 0 | 0 |
| Note: IHC-Immunohistochemistry; score: 0 - negative; 1+ [positive IHC stained positive cells are < 25%]; 2+ [positive IHC stained positive cells are > 25%] | | | | |
