# Supplementary material for: Genetic alterations of Keap1 confers chemotherapeutic resistance through functional activation of Nrf2 and Notch pathway in head and neck squamous cell carcinoma
Source: Cell Death Dis. 2022 Aug 9;13(8):696. doi: 10.1038/s41419-022-05126-8 (PMC9363464; doi:10.1038/s41419-022-05126-8)
Supplement: Supplementary file 10 — Supplementary Table S6 [file 41419_2022_5126_MOESM10_ESM.docx]

| Supplementary Table S6. Keap1 and Nrf2 mutations in Nrf2 immunopositive head and neck cancer tumor tissues | | | | | |
| --- | --- | --- | --- | --- | --- |
| No. of patient | Histology | Keap1 expression^1^ | Keap1 sequence alteration | Nrf2 expression by IHC | Nrf2 sequence alteration^2^ |
| HNSCC-1 | Laryngeal | Absent | c.403C>T | Positive | Not detected |
| HNSCC-2 | Oral cavity | Absent | c.1111G>A | Positive | Not detected |
| HNSCC-3 | Oral cavity | Absent | c.1129G>A | Positive | Not detected |
| HNSCC-4 | Oral cavity | Absent | c.1766A>G | Positive | Not detected |
| HNSCC-5 | Laryngeal | Present | Not detected | Positive | Not detected |
| HNSCC-6 | Oral cavity | Present | Not detected | Positive | Not detected |
| HNSCC-7 | Laryngeal | Present | Not detected | Positive | Not detected |
| HNSCC-8 | Laryngeal | Present | Not detected | Positive | Not detected |
| HNSCC-9 | Hypopharyngeal | Present | Not detected | Positive | Not detected |
| HNSCC-10 | Oral cavity | Present | Not detected | Positive | Not detected |
| HNSCC-11 | Laryngeal | Present | Not detected | Positive | Not detected |
| HNSCC-12 | Laryngeal | Present | Not detected | Positive | Not detected |
| HNSCC-13 | Hypopharyngeal | Present | Not detected | Positive | c.241G>C |
| HNSCC-14 | Oral cavity | Present | Not detected | Positive | Not detected |
| HNSCC-15 | Oral cavity | Present | Not detected | Positive | Not detected |
| HNSCC-16 | Oral cavity | Present | Not detected | Positive | Not detected |
| HNSCC-17 | Oral cavity | Absent | Not detected | Positive | Not detected |
| HNSCC-18 | Laryngeal | Present | Not detected | Positive | Not detected |
| HNSCC-19 | Hypopharyngeal | Present | Not detected | Positive | Not detected |
| HNSCC-20 | Hypopharyngeal | Present | Not detected | Positive | Not detected |
| HNSCC-21 | Laryngeal | Present | Not detected | Positive | Not detected |
| HNSCC-22 | Hypopharyngeal | Present | Not detected | Positive | c.145G>A |
| HNSCC-23 | Laryngeal | Present | Not detected | Positive | Not detected |
| HNSCC-24 | Oral cavity | Present | Not detected | Positive | Not detected |
| HNSCC-25 | Oral cavity | Present | Not detected | Positive | Not detected |
| Notes: ^1^Keap1 expression was determined by qRT-PCR. “Present”- refers that Keap1 transcript was detected after 45 cycle after PCR amplification. “Absent”-refers that Keap1 transcript was not detected after same PCR cycle amplification. All reactions were conducted at least three times.  ^2^ DNA sanger sequencing was conducted and the sequence information described in materials methods section in the main text. | | | | | |
