# Supplementary material for: Genetic alterations of Keap1 confers chemotherapeutic resistance through functional activation of Nrf2 and Notch pathway in head and neck squamous cell carcinoma
Source: Cell Death Dis. 2022 Aug 9;13(8):696. doi: 10.1038/s41419-022-05126-8 (PMC9363464; doi:10.1038/s41419-022-05126-8)
Supplement: Supplementary file 11 — Supplementary Table S7 [file 41419_2022_5126_MOESM11_ESM.docx]

| Supplementary Table S7: Determination of Total GSH and enzymatic activity of SOD1, NQO1 and GST in matched HNSCC normal and tumor tissues | | | | | | | | | | | |
| --- | --- | --- | --- | --- | --- | --- | --- | --- | --- | --- | --- |
|  | PT1* | PT2* | PT3* | PT4* | PT5** | PT6** | PT7 | PT8 | PT9 | PT10 | PT11 |
| **GSH Normal** | 12.45 | 14.22 | 16.17 | 5.13 | 18.24 | 2.18 | 16.11 | 10.55 | 11.7 | 2.17 | 6.77 |
| **GSH Tumor** | 26.14 | 5.17 | 43.51 | 21.56 | 13.61 | 10.57 | 36.47 | 23.22 | 31.76 | 19.64 | 8.18 |
|  |  |  |  |  |  |  |  |  |  |  |  |
| **SOD1 Normal** | 5.9 | 10.5 | 12.8 | 3.5 | 39.1 | 3.8 | 12.45 | 30.14 | 18.7 | 22.14 | 12.25 |
| **SOD1 Tumor** | 6.4 | 14.21 | 124.1 | 22.58 | 6.4 | 29.4 | 36.47 | 152.7 | 142.3 | 121.4 | 52.47 |
|  |  |  |  |  |  |  |  |  |  |  |  |
| **NQO1 Normal** | 2.5 | 30.4 | 20.47 | 26.71 | 5.4 | 10.8 | 6.6 | 5.14 | 29.1 | 15.47 | 18.14 |
| **NQO1 Tumor** | 2.9 | 3.5 | 25.69 | 5.37 | 36.14 | 30.48 | 22.14 | 56.1 | 3.4 | 52.17 | 105.1 |
|  |  |  |  |  |  |  |  |  |  |  |  |
| **GST Normal** | 18.24 | 39.3 | 25.11 | 26.17 | 25.52 | 13.17 | 40.5 | 29.51 | 15.22 | 15.44 | 18.14 |
| **GST Tumor** | 33.4 | 45.17 | 74.2 | 81.33 | 39.72 | 19.62 | 39.44 | 50.25 | 30.75 | 86.1 | 36.93 |
| *Patients with somatic *Keap1* mutation; **Patients with *Nrf2* mutation | | | | | | | | | | | |
