# Supplementary material for: Genetic alterations of Keap1 confers chemotherapeutic resistance through functional activation of Nrf2 and Notch pathway in head and neck squamous cell carcinoma
Source: Cell Death Dis. 2022 Aug 9;13(8):696. doi: 10.1038/s41419-022-05126-8 (PMC9363464; doi:10.1038/s41419-022-05126-8)

Supplemental materials

Genetic alterations of *Keap1* confers chemotherapeutic resistance through functional activation of *Nrf2* and Notch pathway in head and neck squamous cell carcinoma

Abu Shadat M Noman[1], Khawlah Qassem[2], Shafiqul Islam[1], Rashed R Parag[1], Mohammed Z Rahman[3], Walid A Farhat[4], Herman Yeger[5], Abdelillah Aboussekhra[2], Bedri Karakas[2], Syed S Islam[2,6*]

Raw western blots for Figures 3/ 5/7

Original Raw Western blots for Figures 3B

Figure 3B Raw uncropped Western blots

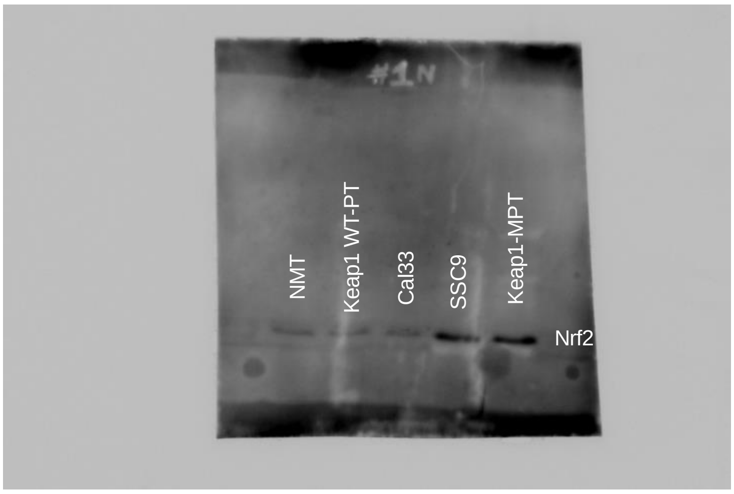

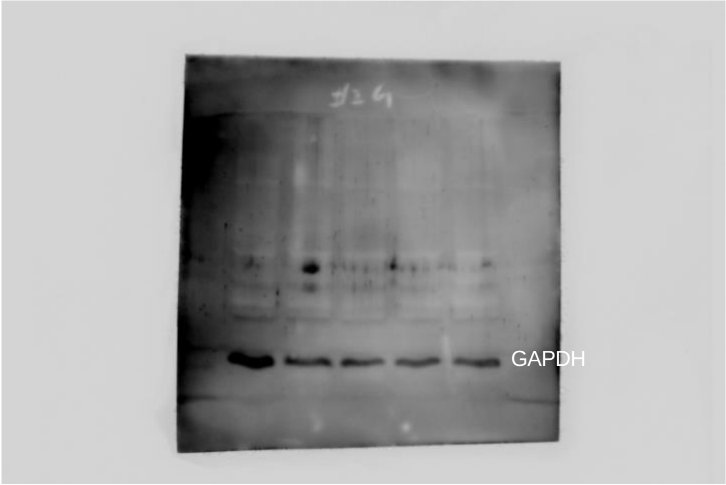

Figure 3B Raw uncropped Western blots

Figure 5A Raw uncropped Nrf2 Western blot

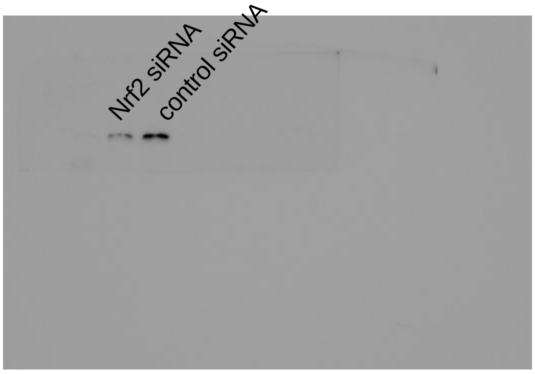

Nrf2

Figure 5A Raw uncropped GAPDH Western blot

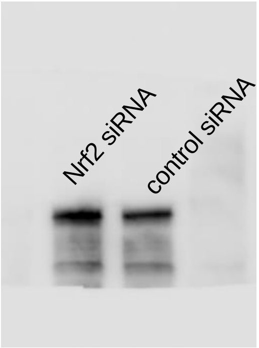

GAPDH

Figure 7B Raw uncropped Notch1 Western blot

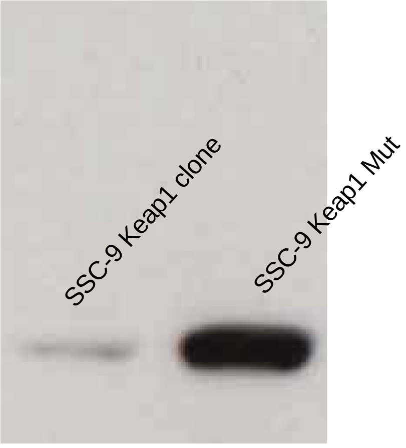

Notch1

Figure 7B Raw uncropped GAPDH Western blot

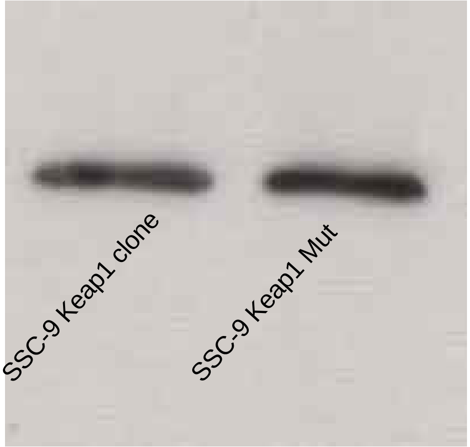

GAPDH

Figure 7B Raw uncropped Hes1 Western blot

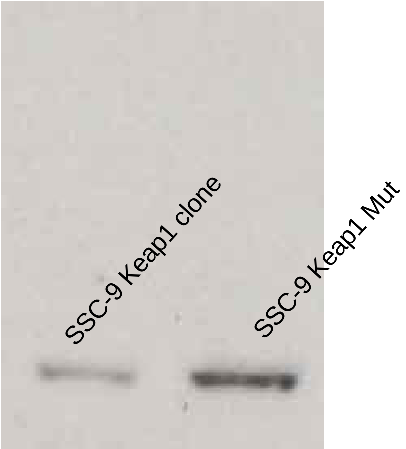

Hes1

Figure 7D Raw uncropped Notch1 Western blot

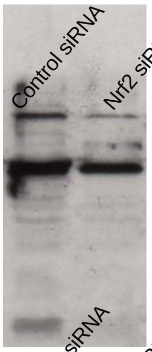

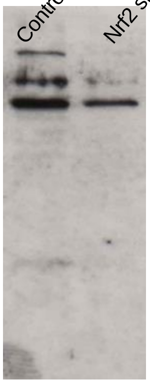

Figure 7D Raw uncropped Hes1 Western blot

Figure 7D Raw uncropped GAPDH Western blot

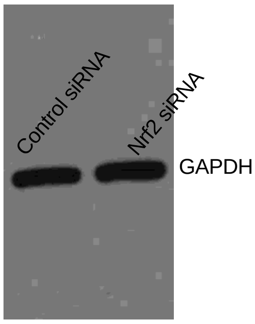

Figure 7F Raw uncropped Notch1 Western blot

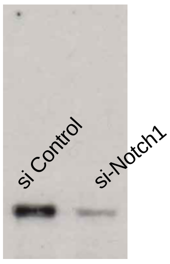

Figure 7F Raw uncropped GAPDH Western blot

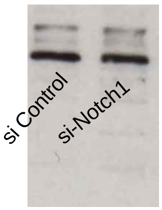

Figure 7I Raw uncropped Hes1 Western blot

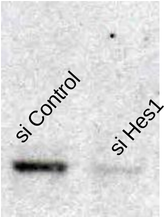

Figure 7I Raw uncropped GAPDH Western blot

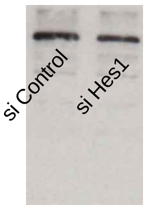

Supplement: Supplementary file 13 — Original Data File [file 41419_2022_5126_MOESM13_ESM.pdf]
